# Functional group classification using consensus clustering

**DOI:** 10.1371/journal.pcbi.1014278

**Published:** 2026-05-13

**Authors:** Pablo Ubilla Pavez, Andrea Paz, Daniel S. Maynard

**Affiliations:** 1 INRIA, Montpellier, France; 2 Department of Genetics, Evolution and Environment, University College London, London, United Kingdom; 3 Département de Sciences Biologiques, Faculté des Arts des Sciences, Université de Montréal, Montréal, Canada; Washington University in Saint Louis, UNITED STATES OF AMERICA

## Abstract

Functional diversity is a fundamental aspect of community structure and composition, reflecting diversity and redundancy in ecological niches, functional roles, and environmental responses among species within a community. Despite its growing importance for quantifying ecosystem-level biodiversity, existing functional diversity metrics remain difficult to calculate and interpret, hindering their adoption and application beyond the scientific realm. One potential solution to this problem is to categorize species into functional groups based on their traits, which provides a simple, intuitive categorization of functional diversity that allows for the application of traditional species-based metrics. The functional-group approach, however, has several challenges that have limited its adoption, namely, the difficulty in identifying robust functional clusters, which can vary substantially due to trait variability, measurement error, and trait correlation. Here, to address these challenges, we present a multi-step consensus clustering method that integrates trait uncertainty and correlation into the classification of species into functional groups. Our approach proceeds in four main steps: (1) (re)sample trait data from an underlying distribution or with measurement error, (2) fit a Gaussian Mixture Model (to account for correlation) to each resample, (3) build a consensus matrix quantifying how often species pairs are grouped together across the noisy trait sample, and (4) apply traditional hierarchical clustering to this matrix and select the final groups. As a case study of this approach, we apply this method to a global dataset of 47,828 tree species using 18 traits, identifying 42 functional groups with distinct trait patterns and varying degrees of stability. We show how the resulting groups reflect underlying ecological trade-offs and phylogenetic structure, and we demonstrate how traditional diversity metrics (richness and Simpson’s Index) can be applied to these functional groups to provide intuitive measures of functional group richness and functional redundancy. Collectively, this framework presents a scalable, interpretable approach for quantifying functional groups that embraces trait correlation and trait uncertainty, allowing for repeatable and intuitive quantification of functional biodiversity that can aid its adoption in biodiversity assessments by conservation and restoration organisations.

## Author summary

Biodiversity is commonly quantified by the number of distinct species within a given ecosystem. In contrast, functional diversity uses traits—such as tree height or leaf area—to quantify how species’ ecological strategies vary. Quantifying functional diversity poses challenges: traits are often correlated, and many species have incomplete trait data. To address this, we introduce a method that generates multiple plausible trait profiles for each species. For each scenario, species are clustered based on trait similarity, and we assess how frequently species are grouped together across scenarios. Species that consistently cluster together are likely to fill similar ecological roles, and use these consensus patterns to form “functional groups” of species that share similar trait profiles. Applying this method to a global dataset of over 47,000 tree species, we identified 42 functional groups, varying in robustness, and we show how these groups relate to underlying trait patterns. Finally, we demonstrate how these groups can be used to measure functional diversity in a clear and practical way, helping conservation and restoration efforts track and protect biodiversity more effectively.

## Introduction

Quantifying biodiversity is an important task because it helps us define conservation targets, evaluate its change over time in different regions, and understand how it relates to different environmental and human pressures [1–4]. The most common approach to tracking biodiversity is by species richness, which is directly related to phylogeny, and thus the genetic difference between organisms [5–7]. While phylogeny offers important insights into biodiversity, functional diversity presents another valuable perspective by focusing on traits rather than just species [8]. This approach is particularly valuable because traits are closely linked to ecological strategies [9], niches [10,11], and species’ adaptability to environmental changes [12,13]. As we enter an era dominated by big data, the accumulation of extensive trait measurements from various species among different taxonomic groups has led to promising approaches in using these traits as features in predictive models [14]. For example, we can predict how an organism would respond to climate change [15,16], survive in particular habitats [17] or coexist with other species [9,18].

Early efforts to quantify functional diversity primarily focused on functional groups—i.e., sets of species that share traits or ecological roles—which provided tractable means to estimate functional richness via traditional diversity metrics [19,20]. These groupings were often assumed to capture meaningful variation in species’ contributions to ecosystems and provided a convenient way to apply categorical diversity metrics [21]. However, functional group delineation was criticized for its subjectivity [22]: group boundaries were typically determined by researcher discretion and thus varied across studies, hindering efforts to compare across systems or regions. Moreover, groupings can obscure the continuous nature of functional traits, limiting the resolution of trait-based ecological inference at smaller spatial or temporal scales [23]. In response, the field has increasingly embraced continuous trait-based approaches, where species are positioned in a multi-dimensional trait space to capture fine-scale variation [24,25]. These methods allow richer, quantitative representations of functional composition and facilitate metrics such as functional dispersion and trait divergence.

Yet continuous trait-based metrics bring their own challenges. As trait dimensionality increases, data become complex and interpretation becomes more difficult [26,27]. Ecologists must choose which traits to include, how to weight them, which of the dozens of trait metrics to use, and how to manage missing data and trait uncertainty—all of which can affect the results and inference, hindering comparisons across studies [25]. More problematically, many trait-based metrics are difficult to interpret for non-specialists [22], constraining their use primarily to academic research, and preventing their adoption in conservation frameworks that rely on simpler, threshold-based decision tools. As such, most biodiversity frameworks largely ignore functional diversity [28–30], reflecting the difficulty in obtaining simple, repeatable, and interpretable metrics that hold across studies.

A return to functional group-based approaches offers several compelling advantages, particularly for broad-scale or policy-oriented biodiversity assessments. Grouping species by shared ecological roles, life-history strategies, or resource-use traits provides a natural means of abstraction that simplifies trait data without necessarily discarding its ecological meaning [21,22,31]. Functional groups are more intuitively interpretable and lend themselves to categorical metrics, decision thresholds, and standardized reporting formats that are already used in conservation planning [30]. Moreover, compared to continuous trait metrics, functional classifications can be more robust to missing data, trait inclusion (or exclusion), and taxonomic inconsistencies [32–35], allowing for broader taxonomic and geographic coverage in global biodiversity datasets. From a practical perspective, functional groups are particularly appealing because biodiversity metrics designed for quantifying species diversity can be directly applied to these functional groups [36,37]. In addition, comprehensive lists of functional groups could be constructed for the use of non-experts, helping with evidence-based assessments of functional diversity.

In order to apply functional group approaches, we need to develop robust methods for classifying species to ensure that trait groupings are informed by underlying traits, and not simply expert knowledge. A clustering algorithm finds patterns in the data by grouping observations in the most fitting way possible based on certain assumptions [38,39]. As clustering is an unsupervised task, there is no agreed-upon correct way of knowing which algorithm would suit a particular dataset [40]. When choosing to use clustering algorithms, it is important that the data is clearly understood and what the reason to cluster is, so that the chosen algorithm aligns with the research question. There are various clustering algorithms with different assumptions. Flat clustering algorithms, also known as partition clustering, group species without creating inter-group structures, while hierarchical clustering algorithms create a hierarchy of groups, allowing for group joining or separation [41]. Clustering algorithms can also be parametric, assuming parameters to model cluster distribution, or non-parametric, identifying clusters based solely on data we observe [42]. In trait ecology, these assumptions impact the ecological interpretation of groups. A flat parametric algorithm suggests each functional role lies somewhere in trait-space and species are driven to these functional roles. A density-based approach suggests species with similar ecological functions evolve their traits systematically, occupying a feasible trait-space for that function.

Regardless of the clustering method, there are two key challenges when clustering functional data: (1) the ubiquity of missing data and data uncertainty, and (2) the curse of dimensionality and correlation. First, most clustering algorithms require a complete trait dataset, which leads to the use of imputation methods for missing trait data via methods such as BHPMF [43] and MissForest [44]. Regardless of the method used for imputation, there is uncertainty introduced to the dataset, as the real value remains unknown and the imputed values have intrinsic imputation error (which is often not reported). Second, because traits are often highly correlated with each other, the addition or removal of specific traits can lead to divergent clustering patterns, compromising some distance metrics and performance. To address the issue, researchers commonly use techniques like PCA [45,46] or recently popularized autoencoders [47,48] to reduce dimensionality and provide orthogonal trait inputs. These methods can be particularly useful in high-dimensional settings (e.g., audio or image classification), but, by favoring traits that capture significant variation across the dataset, they may erase patterns or ignore unique traits that are important for identifying particular groups.

Here, we address these issues by presenting a novel multi-step clustering approach for calculating functional groups, which addresses the combined challenges of trait uncertainty and trait correlation (or trait selection). First, to address uncertainty, our approaches relies on resampling the data (by adding measurement error or by sampling from a known distribution reflecting trait variation), where each resample is interpreted as a possible realisation of the underlying distribution. Second, for each resample, we fit a Gaussian Mixture Model (GMM), which addresses high-dimensionality and collinearity by modelling trait covariation, and we identify the number of components that minimizes the Bayesian Information Criterion (Clustering ensemble). This two-step approach results in a clustering ensemble, which we then synthesize using consensus clustering by computing how often each pair of species was linked together (Consensus clustering). These proportions are then treated as a distance metric between species, making it suitable for the use of hierarchical clustering as a final step in the analysis workflow to obtain a single overall group assignment.

To illustrate this approach, we provide a case-study of 47,828 tree species across the globe, classifying them into functional groups using 18 traits related to leaf, crown, root, seed, stem, and wood physiology and morphology. We show that this approach not only identifies the stable functional groups but also provides insight into the underlying functional traits driving these patterns. By comparing this functional group metric to existing species diversity metrics, we illustrate how functional groups provide an intuitive alternative to more complex functional diversity metrics, allowing us to apply species-based metrics (e.g., Simpson’s Index) to functional group classification. Collectively, the approach developed here presents an adaptable framework that can be applied to other taxonomic groups and geographic regions, making it a promising tool for future biodiversity assessments and ecological studies that require simple, repeatable, and robust measures of functional diversity.

## Materials and methods

### Overview

**Fig 1:**
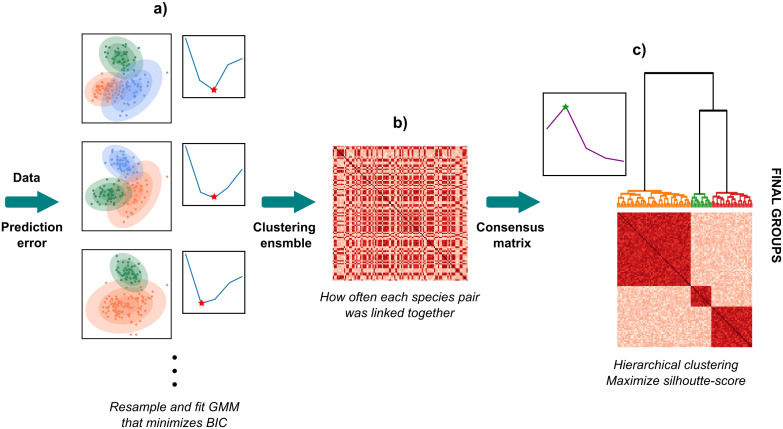
Conceptual diagram for consensus clustering with data uncertainty. **a)** The input of the model is the data and prediction error to account for data uncertainty. These data and prediction error are used to perform resamples that are clustered using GMM by optimizing the number of groups with BIC. **b)** These resamples yield a clustering ensemble, where for each pair of species it is possible to compute the proportion of times they were linked together. This builds a consensus matrix. **c)** This consensus matrix is used as a distance matrix to perform hierarchical clustering to optimize the silhouette score (due to the absence of a likelihood for BIC-based selection) and obtain the final group assignments.

Motivated by the original application of consensus clustering [49], our methodology comprises a multi-step clustering approach that addresses the challenge of uncertainty and correlation between traits (Fig 1). In summary, we resample our dataset *S* times, generating a clustering ensemble, where, for each resample, we fit a GMM with the total number of components (functional groups) that minimizes BIC (Clustering ensemble), such that every species is assigned to a group which can vary within iterations. This generates a clustering ensemble that is synthesized using consensus clustering by computing how often each pair of species was linked together (Consensus clustering). These proportions are then treated as a distance metric between species, making it suitable for the use of hierarchical clustering. This allows for a flexible clustering scheme, where the number of groups can be decided based on the particular application.

Throughout, we denote matrices with bold and capital letters (e.g., ***A***), vectors with bold and lowercase letters (e.g., ***a***), and scalars and indices with non-bold letters (e.g., *a*, *i*, *j*). The cardinality of a set 𝒮, representing the number of elements in the set, is denoted by |𝒮|. We otherwise define any notation that is specific to this methodology.

### Clustering ensemble

The first step in this method addresses the fact that trait data are noisy, whether due to intraspecific or ontogenetic variation, or to measurement error or imputation noise. We address this uncertainty by adopting an ensemble approach to clustering, where we resample the data in the presence of uncertainty, and fit a Gaussian mixture model to each resample.

Specifically, let ***X*** denote a matrix in which each row corresponds to a species, and each column represents a trait. Formally, ***X*** has *N* rows (species) and *M* columns (traits). Let us consider 𝒩 as the set containing all species, and ℳ as the set containing all traits. We are not considering the case where some traits could be categorical, as this further complicates the clustering problem [50].

The clustering problem itself consists of finding a partition of 𝒩, denoted as 𝒫, containing a collection of sets that do not share any element with each other and that would jointly account for every species in 𝒩. We denote *G* as the number of sets from the partition, which is equivalent to the number of groups. Also, denote ${𝒞}_{g}$ as the set *g* in the partition. The first challenge of this problem is that we do not know the value of *G*, as this is part of what we are trying to answer.

The second challenge of our clustering problem is that our matrix of observations ***X*** comes with uncertainty. This happens because some entries of the matrix were imputed using predictive methods. To address this uncertainty, we are going to consider the input of the clustering problem to be a random variable ***W***, which we can resample *S* times. Then, let ***W***^(*s*)^ represent the *s*-th instance for clustering, where these instances are independently and identically distributed. Each resampling iteration yields a distinct partition ${𝒫}^{\left(s\right)}$, which may come with a different total number of groups *G*^(*s*)^.

In this section, we will first detail how to model the random variable ***W***, then detail how to fit a GMM for each resample of this variable, and finally how to choose the best value of *G* for each resample.

#### Resampling from error distribution.

To account for trait variation and prediction errors in our data, we use a resampling approach that is repeated in order to obtain a robust cluster. Think of *W*_*ij*_ as a random variable for the value of trait *j* for species *i*, with the following distribution:


$$\begin{array}{c} W_{i,j}=X_{i,j}+\epsilon_{ij}\;\;\epsilon_{ij}\sim E_{ij}\;\;\forall i\in \{1,...,N\},\;j\in \{1,...,M\} \end{array}$$ (1)

Here, *E*_*ij*_ denotes the error distribution for species *i* and trait *j*. For example, this can be specified non-parametrically by sampling from empirical prediction errors, or parametrically using a normal distribution centered at zero with the observed standard deviation. The matrix ***W*** is then a random matrix, where each entry represents a random variable reflecting uncertainty in trait values.

### Fitting a Gaussian mixture model

We adopt to use Gaussian Mixture Models for each resample because of the intrinsic ability of a GMM to account for correlation between traits, and its ability to allow for different correlation structures for each group [51]. Moreover, GMMs provide a flexible probabilistic framework for clustering that can capture a wide range of cluster shapes through mixtures of Gaussian components. Compared to methods such as k-means or PAM, which rely on distance-based partitioning, GMMs allow clusters to differ in covariance structure and orientation. This flexibility is particularly useful for ecological trait data. In particular, the data used in our experiments exhibit a clear correlation structure, and the traits are approximately normally distributed (S1 and S2 Figs), making GMM a well-suited choice in this case. Nevertheless, depending on the ecological application and the expected importance of trait covariation, alternative clustering methods may be more appropriate.

For the effects of this section, consider W=w to be any realization of the random variable that serves as input for the clustering algorithm. Note that in cases with uncertainty in the data, fitting a GMM without resampling likely leads to overfitting (S10 Fig).

In a GMM, each group *g* would be defined by a Multivariate Normal distribution with mean $\mu_{g}$, covariance matrix $\Sigma_{g}$, and weight $\pi_{g}$, that determines the probability of a species belonging to group *g*. We will refer to the complete set of parameters as: $\theta^{\left(G\right)}=\{\mu_{g},\Sigma_{g},\pi_{g}{\}}_{g=1}^{G}$. These parameters are latent variables of the model (unobserved) that we are trying to recover by maximizing log-likelihood.

To estimate the parameters $\theta^{\left(G\right)}$, we maximize the log-likelihood using the EM-algorithm [52], which involves two steps:

**E-step:** Calculate the posterior probabilities $\tau_{ig}$ that *i* belongs to *g*, given the current parameter estimates. Usin*g* Bayes and the law of total probability:


$$\begin{array}{cc} \tau_{ig} & =P(i\in {𝒞}_{g}|W_{i}=w_{i};\theta^{\left(G\right)})\;\;\forall i\in 𝒩\;\forall g\in \{1,...,G\}\\ & =\frac{P(W_{i}=w_{i}|i\in {𝒞}_{g};\theta^{\left(G\right)})\cdot P(i\in {𝒞}_{g};\theta^{\left(G\right)})}{P(W_{i};\theta^{\left(G\right)})}\\ & =\frac{\phi (W_{i}=w_{i};\mu_{g},\Sigma_{g})\cdot \pi_{g}}{\sum_{h=1}^{G}\phi (W_{i}=w_{i};\mu_{h},\Sigma_{h})\cdot \pi_{h}} \end{array}$$ (2)

where ϕ is the Multivariate Normal density function.

**M-step:** Update the parameters $\mu_{g}$, $\Sigma_{g}$, and $\pi_{g}$ by maximizing the expected log-likelihood (3) using the posterior probabilities $\tau_{ig}$ from the E-step (2).


$$\begin{array}{c} l(\theta^{\left(G\right)};W=w)=\sum_{i=1}^{N}\ln\left(\sum_{g=1}^{G}\pi_{g}\phi (W_{i}=w_{i};\mu_{g},\Sigma_{g})\right)\;\;\forall g\in \{1,...,G\} \end{array}$$ (3)

The algorithm alternates between these steps until the parameter estimates converge. After fitting the model, group membership for each species can be interpreted either as a probability distribution, where $P(i\in {𝒞}_{g})=\tau_{ig}$, or by assigning each species to the group with the highest posterior probability, $i\in {𝒞}_{g}|g=\arg\max_{g}\tau_{ig}$.

Choosing the optimal number of groups *G* is challenging since increasing *G* naturally improves the log-likelihood by adding more parameters. Thus, the goal is to find the smallest *G* that still adequately fits the data.

An approach to choose the value of *G* is to use the Bayesian Information Criterion (BIC), as we are working directly with a probability distribution. This metric balances the trade-off between the log-likelihood and the number of parameters. This metric has been shown to estimate the correct true number of groups in multiple situations under certain simulated conditions and is usually recommended against other metrics like the Akaike Information Criterion [53]. Note that the number of parameters increases linearly as we fit more groups. Defining $\left|\theta_{unique}^{\left(G\right)}\right|$ as the number of parameters for a GMM with *G* groups (without considering repeated parameters), the BIC value for a GMM with *G* components is:


$$\begin{array}{c} |\theta_{unique}^{\left(G\right)}|=\underset{\text{Number of weights}}{\underbrace{\left(G-1\right)}}+\underset{\text{Number of means}}{\underbrace{\left(G\cdot M\right)}}+\underset{\text{Number of covariances}}{\underbrace{\left(G\cdot M\cdot (M+1)\cdot 2^{-1}\right)}} \end{array}$$ (4)

$$\begin{array}{c} BIC_{G}=-2\cdot l(\theta^{\left(G\right)};X)+|\theta_{unique}^{\left(G\right)}|\cdot \ln\left(N\right) \end{array}$$ (5)

Using this metric, we can try different values of *G* and choose the GMM that minimizes the BIC score. Then, we can define $G^{*}=\arg\min_{G}BIC_{G}$. Note that other metrics used for choosing the number of clusters, such as the silhouette-score or the elbow method, do not penalize model complexity, can be affected by correlation, and ignore the probabilistic nature of the model [53–55].

### Consensus clustering

Through resampling, we obtained a collection of partitions ${𝒫}^{\left(1\right)},{𝒫}^{\left(2\right)},...,{𝒫}^{\left(S\right)}$, each of which is the best clustering result for a particular resample of our data. This is useful, as we generated a distribution of clusters that accounts for uncertainty. However, this process introduces a new challenge: determining a singular assignment for each species from the multitude of individual clusterings. To address this issue, we need to decide a singular assignment for species *i* based on how often it was linked to the other species $i^{\prime }\neq i$.

The idea of consensus clustering was first introduced for visualizing gene expression [49]. The main purpose of this algorithm is to find consensus between multiple cluster algorithms or multiple realizations of the same algorithm. While the original framework proposes bootstrapping as a resampling method, we saw the potential to incorporate prediction error as a resampling mechanism to address uncertainty. Then, we construct a consensus matrix ***C*** that summarizes the clustering ensemble previously obtained. In this matrix, component $C_{ii^{\prime }}$ shows how often species *i* was linked to species $i^{\prime }$ throughout the *S* iterations of resampling.


$$\begin{array}{c} C_{ii^{\prime }}=\sum_{s=1}^{S}\frac{\sum_{g=1}^{G^{\left(s\right)}}1\{(i\;\&\;i^{\prime })\in {𝒞}_{g}^{\left(s\right)}\}}{S}\;i\neq i^{\prime } \end{array}$$ (6)

$$\begin{array}{c} C_{ii}=1 \end{array}$$

Note that the clustering ensemble and the construction of the consensus matrix could be done simultaneously in an efficient way as described in Algorithm 1.

In this section, we will go through how to build a clustering structure with the consensus matrix, and how to ultimately choose the number of groups with this structure.

**Algorithm 1 Consensus Clustering with Trait Resampling and GMM random assignments**

1:  **Input:** Original matrix $X\in {\mathbb{R}}^{N\times M}$, error distributions *E*, number of samples *S*, number of random assignments *A*

2:  **Output:** Final matrix ***C*** containing average cluster co-occurrences

3:  Initialize ***C*** as an *N* × *N* zero matrix

4:  **for** *s* = 1 to *S* **do**

5:   Sample ***W***^(*s*)^ as described in (1)

6:   Fit GMM to ***W***^(*s*)^ to obtain probability matrix $\tau^{\left(s\right)}$ as described in (2)

7:   **for** *a* = 1 to *A* **do**

8:    Sample partition ${𝒫}^{\left(s,a\right)}$ from $\tau^{\left(s\right)}$

9:    Initialize $C^{\left(s,a\right)}$ as an *N* × *N* zero matrix

10:    **for** each pair $\left(i,i^{\prime }\right)$ **do**

11:     $C_{i,i^{\prime }}^{\left(s,a\right)}\leftarrow 1$ if $\exists 𝒞\in {𝒫}^{\left(s,a\right)}|(i\&i^{\prime })\in 𝒞$, otherwise 0

12:    **end for**

13:    $C\leftarrow C+C^{\left(s,a\right)}$

14:   **end for**

15:  **end for**

16:  $C\leftarrow \frac{1}{S\cdot A}C$

### Hierarchical clustering

Hierarchical clustering, particularly linkage-based methods, is well-suited for our clustering framework because it requires only a distance metric rather than specific observations. This is ideal for our case, as the consensus matrix ***C*** can be interpreted as a distance matrix. By defining D=1−C, we treat species with high consensus as closer to each other for clustering purposes, making hierarchical clustering a natural choice for this step.

For our application, we employ the Ward linkage method due to its ability to create compact, homogeneous clusters while minimizing variance [40], making it widely adopted in ecological studies for phylogenetics and species distribution [49,56,57]. It forms clusters by merging pairs that result in the smallest increase in within-cluster variance at each step using an agglomerative approach. In doing so, it implicitly results in clusters that are compact and approximately spherical in multivariate trait space, which is consistent with ecological niche theory, which holds that species occupy bounded, unimodal regions in multidimensional niche and trait space [58,33]. Nevertheless, although we adopt Ward’s method due to its simplicity and ecological relevance, we stress that any hierarchical clustering linkage method could be used in place, depending on the desired application and resolution of small vs. large clusters, and the expected shape of the ecologically relevant clusters in multivariate trait space.

#### Choosing the number of groups for hierarchical clustering.

As with the GMM procedure, we once again face the challenge of choosing the number of groups to obtain a single consensus cluster. We address this using a dendrogram generated by the Ward linkage, which is a tree-like diagram that illustrates the arrangement of clusters formed at various levels. We denote the number of groups as *K* to distinguish it from those in the GMM. Unlike GMM, which is a flat clustering algorithm, hierarchical clustering allows flexibility to adjust *K* by cutting the dendrogram at different levels [59].

To choose the best value for *K*, we use the silhouette score, which measures how well a species aligns with its cluster versus other clusters. This score is effective in identifying the true number of clusters across various applications [54]. In contrast to the previous resampling step, which used a likelihood-based criterion such as BIC, this approach relies on a distance matrix and therefore does not permit the use of likelihood-based methods. The silhouette score *s*_*K*_(*i*) for species *i* in a clustering with *K* groups is defined as:


$$\begin{array}{c} s_{K}(i)=\frac{b_{K}(i)-a_{K}(i)}{\max\{a_{K}(i),b_{K}(i)\}}\;\forall i\in 𝒩 \end{array}$$ (7)

$$\begin{array}{c} a_{K}(i)=\frac{1}{|{𝒞}_{k(i)}|-1}\sum_{i^{\prime }\in {𝒞}_{k(i)},i^{\prime }\neq i}(1-C_{ii^{\prime }}) \end{array}$$

$$\begin{array}{c} b_{K}(i)=\min_{{𝒞}_{k(i)}\neq {𝒞}_{k^{\prime }}}\frac{1}{\left|{𝒞}_{k^{\prime }}\right|}\sum_{i^{\prime }\in {𝒞}_{k^{\prime }}}(1-C_{ii^{\prime }}) \end{array}$$

Where intuitively: *a*_*K*_(*i*) is the average distance from species *i* to all other species in the same cluster, and *b*_*K*_(*i*) is the average distance from species *i* to all the species in the nearest next cluster. Then K^*^ is the value that maximizes $\sum_{i=1}^{N}s_{K}(i)$.

By maximizing the silhouette score, we obtain a final partition of 𝒩, denoted as ${𝒫}_{K^{*}}$, which serves as the outcome of the clustering framework. Note again that this partition is hierarchical, not flat like the one used for GMM, allowing flexibility to adjust the number of groups ($K<K^{*}$ or $K>K^{*}$) within the same clustering structure.

It is important to note that the optimum in terms of silhouette-score comes from a hierarchical clustering perspective that is different from the resampling approach. The optimal number of clusters chosen at the last step does not need to match the mean of optimums obtained during the resampling step.

### Case study: Functional groups of trees

Traits are particularly important for trees, as they govern water, nutrient, and light economies within individuals [1,60], which underpin key life-history trade-offs that lead to trait selection across different environments [61]. Trees have unique traits related to woody structure, size, and longevity [62,63], which sets them apart from herbaceous, non-dendritic plant species, whose analysis is often restricted to leaf-related traits [64,65]. The unique traits of trees can provide insight into broader ecological functions of trees, such as a forest’s carbon storage capacity [66,67] and its resilience to climate change [15,68,69]. Such analyses are indispensable for predicting the impacts of environmental shifts and planning effective conservation strategies.

In this case study, we apply our consensus clustering approach in the presence of trait uncertainty, as some of the trait measurements have been imputed using machine learning methods [70]. We address two main questions: (1) how many groups there are, and (2) what these groups are. Question (1) is addressed from an optimization perspective, where we maximize metrics that are related to how good a clustering result is. Question (2) is addressed from a perspective of trait uncertainty, where each resample of the data yields a different possible clustering.

#### Data.

The dataset for this case study is based on observed species-level values and imputed values from a study analyzing global tree trait relationships [70]. Imputed values were generated using machine learning methods based on the phylogenetic distances between species, which introduces the uncertainty we will address in the clustering problem. The data consists of *N* = 47,828 species of trees and *M* = 18 traits (for details see Table 1, S1 and S2 Figs). This data accounts for around 70% of all tree species, across different taxa and geographic regions [71].

**Table 1:** Trait statistics. Each trait specifies the unit of measurement, the mean and standard deviation for natural-log transformed values, the mean, standard deviation, minimum, and maximum values for the original scale, the number of error samples, and the Mean Absolute Error (MAE) on the standardised log scale.

Trait name	*ln*()		Original Scale				N. Errors	MAE (log)
	Mean	Std. Dev.	Mean	Std. Dev.	Min	Max		
Bark thickness (*mm*)	1.21	0.78	4.64	4.76	0.35	42.70	1,085	0.405
Crown diameter (*m*)	2.39	0.49	12.38	7.36	2.84	40.00	–	–
Crown height (*m*)	2.42	0.42	12.29	5.39	3.50	41.30	–	–
Leaf K per mass $\left(\frac{mg}{g}\right)$	2.14	0.42	9.29	3.92	1.04	27.43	1,511	0.242
Leaf N per mass $\left(\frac{mg}{g}\right)$	2.91	0.30	19.23	5.64	4.72	43.50	4,538	0.099
Leaf P per mass $\left(\frac{mg}{g}\right)$	-0.02	0.43	1.08	0.47	0.16	3.90	3,018	0.199
Leaf Vcmax per dry mass $\left(\frac{\mu mol}{g\cdot s}\right)$	-1.17	0.37	0.33	0.12	0.08	0.92	527	0.261
Leaf area (*cm*^2^)	7.79	1.48	5,693.45	8,526.82	6.64	108,949.93	699	0.793
Leaf density $\left(\frac{g}{cm^{3}}\right)$	-1.03	0.26	0.37	0.08	0.04	0.74	1,488	0.160
Leaf thickness (*mm*)	-1.51	0.31	0.23	0.09	0.06	1.21	1,847	0.153
Root depth (*m*)	0.95	0.88	4.19	6.51	0.46	60.00	–	–
Seed dry mass (*mg*)	4.05	2.42	522.19	1,638.89	0.09	41,832.44	5,204	0.863
Specific leaf area $\left(\frac{cm^{2}}{g}\right)$	2.45	0.38	12.37	4.52	1.53	38.46	4,627	0.143
Stem conduit diameter (μm)	3.36	0.32	30.43	10.29	8.92	72.70	252	0.189
Stem diameter (*m*)	-0.61	0.55	0.64	0.45	0.15	6.48	–	–
Stomatal conductance $\left(\frac{mmol}{m^{2}\cdot s}\right)$	5.03	0.46	170.90	85.51	25.42	715.19	866	0.334
Tree height (*m*)	3.23	0.41	27.44	11.56	6.26	119.80	–	–
Wood density $\left(\frac{g_{\text{dry}}}{cm_{\text{wet}}^{3}}\right)$	-0.55	0.23	0.59	0.13	0.18	1.16	7,224	0.103

Prior to clustering, all traits were log-transformed, as is typical for functional trait analysis in plants [72]. This is for two reasons: first, ecologically meaningful differences in traits are typically proportional rather than absolute, as strategies are differentiated by orders of magnitude rather than additive differences. In addition, because most ecological traits are strongly right-skewed, the log transformation stabilizes the variance and ensures approximate normality, which satisfies the assumptions of Gaussian processes.

#### Error data.

In the case of trees, the error distribution for trait *j* was computed by collecting all empirical errors available for that trait (S3 Fig). Maynard et al. produced species-level mean estimates of 18 traits, along with the corresponding error (observed - predicted) for each species that had one or more observed trait values. By collecting these trait-specific errors, we obtain an empirical posterior error distribution for each trait, nested within taxonomic groups (i.e., one set for angiosperms, one set for gymnosperms).

Specifically, let ${ℐ}_{j}^{\left(obs\right)}$ and ${ℐ}_{j}^{\left(pred\right)}$ be the sets of observed and predicted values for trait *j*, respectively. We also differentiate between a set of angiosperms 𝒜 and a set of gymnosperms 𝒢, as these taxonomic groups are significantly different and some methodologies to measure their traits could vary [73]. We specifically have |𝒜|=47,263 angiosperms species and |𝒢|=565 gymnosperm species. Then for traits that we know some observed values, we can sample errors using the following error sets:


$$\begin{array}{c} {ℰ}_{𝒜j}=\{X_{ij}^{\left(pred\right)}-X_{ij}^{\left(obs\right)},\;\forall i\in \left({ℐ}_{j}^{\left(obs\right)}\cap 𝒜\right)\}\;E_{𝒜j}=Uniform({ℰ}_{𝒜j})\;\forall j:|{ℐ}_{j}^{\left(obs\right)}|>0 \end{array}$$ (8)

$$\begin{array}{c} {ℰ}_{𝒢j}=\{X_{ij}^{\left(pred\right)}-X_{ij}^{\left(obs\right)},\;\forall i\in \left({ℐ}_{j}^{\left(obs\right)}\cap 𝒢\right)\}\;E_{𝒢j}=Uniform({ℰ}_{𝒢j})\;\forall j:|{ℐ}_{j}^{\left(obs\right)}|>0 \end{array}$$

Note that for some traits we do not have error samples (marked empty in Table 1). This happens because these traits were predicted using quantile random forest [70], therefore the observed and predicted values are not comparable for these traits. For this case, we assume that the error distribution comes from the union of all sets of errors that we do have.


$$\begin{array}{c} {ℰ}_{𝒜j^{\prime }}=\underset{j\in {ℳ}^{\left(obs\right)}}{⋃}{ℰ}_{𝒜j}\;\;\forall j^{\prime }:|{ℐ}_{j^{\prime }}^{\left(obs\right)}|=0 \end{array}$$ (9)

$$\begin{array}{c} {ℰ}_{𝒢j^{\prime }}=\underset{j\in {ℳ}^{\left(obs\right)}}{⋃}{ℰ}_{𝒢j}\;\;\forall j^{\prime }:|{ℐ}_{j^{\prime }}^{\left(obs\right)}|=0 \end{array}$$

This configuration of errors is well-suited for this particular case study, but it is recommended to adopt an error distribution that suits the particular application. In our case study, for example, we do not consider correlations between errors, in part because the sparsity of the original data set means that the covariance matrix cannot be robustly calculated apart from a subset of species [70]. We thus assume uncorrelated errors in our approach, with the prediction errors being sampled directly from the posterior empirical error distribution for each trait. In other settings, however, more complex error distributions could be incorporated, such as with Bayesian extension of probabilistic matrix factorization (BHPMF), which has been used to impute missing data in other plant datasets [43]. Such decisions should be based on the data availability and the question of interest.

#### Model configuration.

For GMM fitting, we consider *S*=50 resamples of the data, as this yields a reasonable standard error (∼0.07) given the computational limitations. As with any bootstrap or resampling procedure, one should generally take the maximum number of resamples possible, given computational resources and time availability. For each resample, we look for the optimum between *G* = 10–60. For the hierarchical clustering step we test values *K* = 2–120. These ranges were chosen to reflect the values observed in our experiments, where the optimal solutions consistently fell within the mid-range, indicating that at least a local optimum had been identified.

To test different configurations of our framework, we varied two components: (1) feature scaling, and (2) group assignment. For (1) feature scaling, we compare (1.i) the original log-scale values (as described in Data), and (1.ii) a robust scaling approach, where each trait column was centered by its median and scaled by its interquartile range—that is, the difference between the 75th and 25th percentiles. For (2) group assignment (see Fitting a Gaussian Mixture Model), we evaluated (2.i) a sampling-based strategy, where group membership for each species was drawn from the probabilities $\tau_{ig}$, using *A* = 10 assignments per realization of ***W***, and (2.ii) a deterministic strategy, where each species was assigned to its most probable group by taking the maximum of $\tau_{ig}$. Among the resulting four configurations, we selected the one that maximizes the final framework metric: the silhouette score.

#### Analysis.

Using the final clustering partition ${𝒫}_{K^{*}}$ derived from the global tree dataset, we conducted a series of downstream analyses to visualize the resulting clusters, evaluate their stability, examine trait composition and variation within and among clusters, and compare functional biodiversity metrics based on this clustering approach with those obtained from existing methods.

### Visualization

To visualize the clustering results, we use the following dimensionality reduction methods:

PCA [46]: Linear dimensionality reduction by creating new, uncorrelated variables ordered by variance.

t-SNE [74]: Uses a probabilistic approach to match a lower-dimensional distribution to the original.

UMAP [75]: Matches graph structures between high and low dimensions capturing non-linear relations.

It is important to acknowledge that dimensionality reduction methods may result in the loss of some information; however, they are useful to allow human interpretability of the data patterns.

### Statistics

For each cluster ${𝒞}_{k}\in {𝒫}_{K^{*}}$, we computed aggregate statistics, such as the mean and standard deviation of each trait across species $i\in {𝒞}_{k}$. Additionally, we analyzed the cluster sizes $\left|{𝒞}_{k}\right|$ and examine the dendrogram that produces the partition.

For species level stability we explored how similar a species is to other species in its own cluster, and how similar it is to all species overall. This is expressed as:


$$\begin{array}{c} stab_{i}=\frac{\sum_{i^{\prime }:i^{\prime }\in {𝒞}_{k(i)},i^{\prime }=\;\;/\;i}C_{ii^{\prime }}}{\sum_{i^{\prime }=\;\;/\;i}C_{ii^{\prime }}} \end{array}$$

We evaluated clustering robustness by comparing our consensus-based approach to repeated Gaussian Mixture Model (GMM) fits under resampling. On a reduced dataset, we generated multiple resampled subsets and computed pairwise Adjusted Rand Index (ARI) scores between resulting clusterings. We then applied our consensus procedure to groups of resampled runs and compared the stability of the resulting consensus clusterings.

When comparing group-based functional diversity metrics to traditional metrics, we use generalized additive models (GAMs) to account for spatial autocorrelation. Specifically, using the *mgcv* package in R, we fit GAMs using a B-spline basis for each predictor (k = 7, second-order penalty with first-order derivative), with a spherical smooth of geographic coordinates to account for spatial autocorrelation. Models were estimated using restricted maximum likelihood (REML) with an elevated smoothing parameter (γ = 1.4) to guard against overfitting (e.g., gam(y ∼ s(x, bs = ‘bs’, k = 7, m = c(2, 1)) + s(Longitude, Latitude, bs = “sos”)). To estimate residual correlation after accounting for spatial autocorrelations, we fit separate GAMs for the predictor and outcome variables, retaining only the geographic spline term. We then calculated the Pearson correlation between the residuals of both models, providing a measure of association between x and y after removing spatially structured variation in both variables.

### Software and hardware

**Fig 2:**
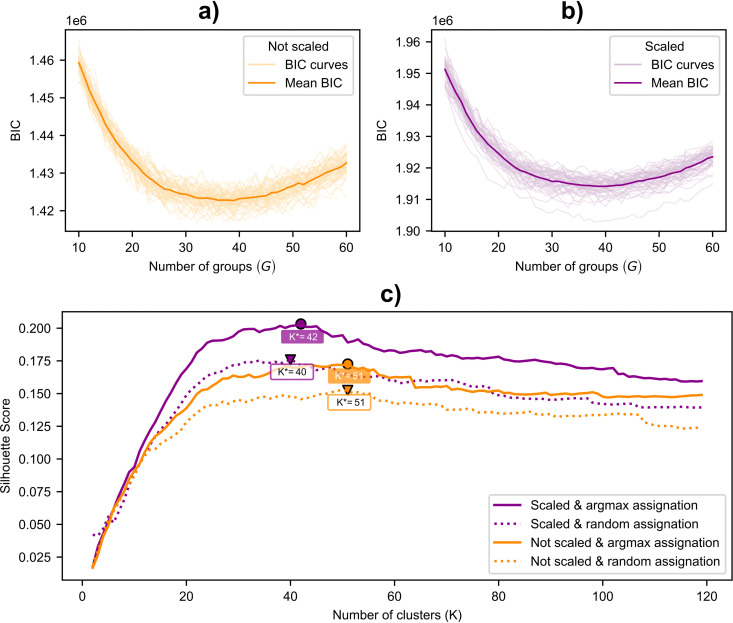
Optimization for number of groups. All BIC curves and the resulting average curve, shown for **a)** unscaled and **b) scaled** data. **c)**: Silhouette score for different aggregations of the consensus matrix. A higher value of *K* means going lower in the dendrogram, hence splitting one group. Note that BIC is optimized independently for each resample in (a) and **(b)**, and therefore partitions with the same number of clusters may differ substantially. In contrast, the Silhouette score is optimized at the aggregated level across all resamples. As a result, the optimal number of clusters identified by these two criteria will not necessarily coincide.

The analysis was primarily done in *Python* 3.12.4, using *scikit-learn* for Gaussian Mixture Models [76], *SciPy* for hierarchical clustering [77], *pandas* for data manipulation [78], *matplotlib* [79], and *seaborn* [80] for visualizations. Computational experiments were conducted on a compute node equipped with 2× Intel Xeon Gold 6426Y (Sapphire Rapids) CPUs (32 physical cores total) and 512 GB of RAM; and initial model testing and downstream analyses were conducted on an AMD Ryzen Threadripper PRO 5995WX workstation (64 physical cores) with 1024 GB DDR4 RAM. The primary experiment (the most computationally demanding and corresponding to Fig 2) required approximately 7.5 hours using the described hardware configuration.

Code is available at https://github.com/pabloubilla/tree_clustering/ and is archived via Zenodo with corresponding DOI:10.5281/zenodo.19222113. The data have been archived via Figshare with corresponding DOI:10.5522/04/31833526.

## Results

### Group selection through consensus clustering

For each individual data resampling, we obtain a corresponding BIC curve that identifies the optimal number of groups (G^*^) at different values (Fig 2). The mean BIC curves exhibit similar patterns for both scaled and non-scaled resamples, although the BIC values are generally lower for the non-scaled resamples. However, since the BIC values come from different distributions, this does not imply that not scaling yields a better fit.

When comparing the four combinations of scaled or not scaled, and argmax or random assignment, we find they display similar patterns by quickly increasing the score until around 30 groups, then reaching a plateau with slow decreases after reaching their respective optima (Fig 2). The highest silhouette score is achieved with 42 groups for the scaled data at 0.20 using the argmax assignment, which was subsequently used for the final group assignment through hierarchical clustering.

Note that a value of 0.20 represents the optimal result given the level of uncertainty in the data; lower uncertainty would be expected to yield higher consensus and correspondingly higher silhouette scores. If all traits had zero error, the consensus would necessarily be 1.0, and if trait noise was substantially greater, the silhouette score would necessarily be lower. Thus, the silhouette score can also be used to indicate the extent to which trait error (or noise) weakens cohesion across the groups.

Using a reduced dataset of 1,365 species, we find that the stability of the clustering results is markedly improved following the consensus step (S4 Fig).

### Functional groups results and visualization

**Fig 3:**
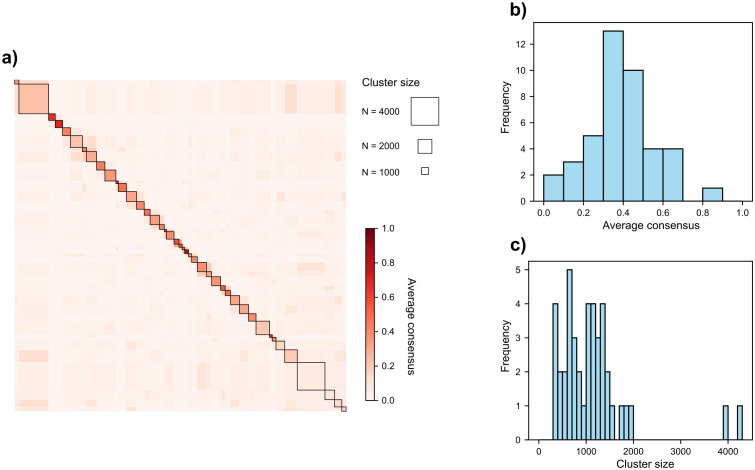
Summary of functional groups results. **(a)** The consensus matrix displays the average consensus for all species pairs within clusters along the diagonal, while off-diagonal values represent the average consensus between species from different clusters (row vs. column). Square sizes are proportional to their respective cluster size. **(b)** Distribution of average consensus for the 42 observed groups, ranging from 0 (no consensus) to 1 (maximum consensus), divided into bins of width 0.1. **(c)** Size distribution of the 42 groups, where cluster size refers to the number of species per group, with bins of width 100.

When grouping species using hierarchical clustering into 42 groups, we observe a wide range of group sizes and varying levels of consensus within the groups (Fig 3). The consensus values are approximately normally distributed, with a mean of 0.39 and a standard deviation of 0.17. Ideally, average consensus values should be near 1, with dissimilarities (off-diagonal values) close to 0, forming a distinct red diagonal in the consensus matrix. We observe a similar result when looking at the species-level stability (S14 Fig). Our results show most clusters tend not to pair with others, except the last six clusters which show uncertain assignments and have average consensus below 0.2 (S1 Table).

The cluster size distribution is left-skewed, with a mean size of 1,139 species and a standard deviation of 790. The majority of clusters contain close to 1,000 species, while a few larger clusters deviate significantly from this norm (Fig 3 and S1 Table). The size distribution is somewhat inconsistent, displaying gaps, which might be due to the relatively low number of clusters and the large range of potential cluster sizes. Notably, there is no cluster smaller than 300 species, with the minimum size being 324 species.

**Fig 4:**
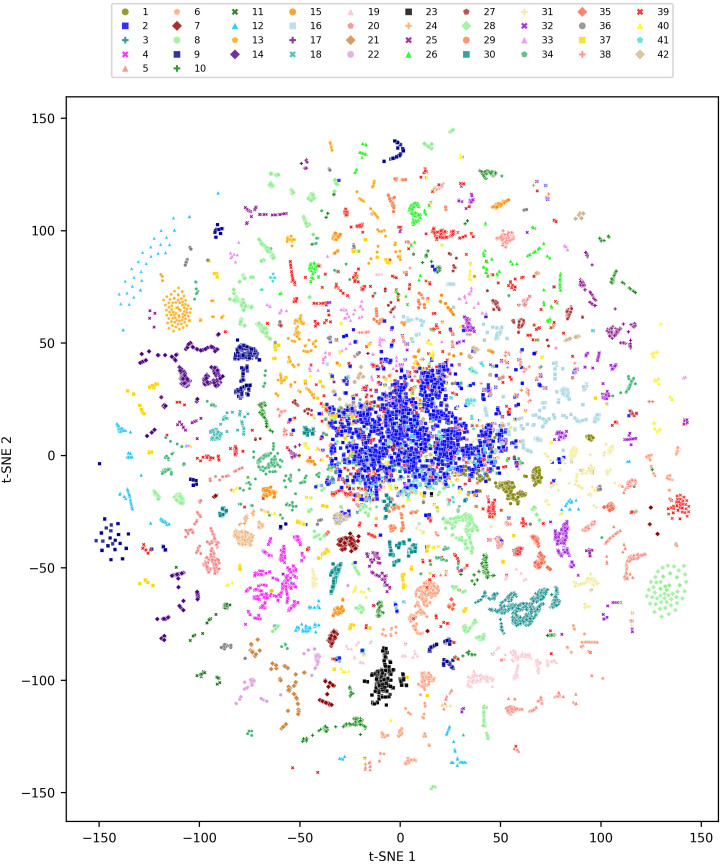
t-SNE visualization of clustering results. Species are visualized in a two-dimensional functional space using t-Distributed Stochastic Neighbor Embedding (t-SNE), a probabilistic technique that reduces high-dimensional data (originally 18 dimensions) to two dimensions while preserving the local structure and similarity relationships of the data points. Different colors and markers represent distinct groups, chosen to maximize visual distinction among clusters based on their distribution. The distances in this 2D space are probabilistic, reflecting the likelihood of similarity between species, and approximate the original high-dimensional probability distribution. Note that t-SNE is intended for visualisation of the shape and location of clusters, but it does not have the same interpretation as other dimensionality reduction techniques such as PCA (see S5 and S6 Figs for alternate visualisations). Please also see the interactive version of this figure in S1 Appendix, or available from the GitHub repository.

We are able to visualize the functional groups using t-SNE (Fig 4, interactive version in S1 Appendix), where we generally observe a clear separation between groups. Group 2 (blue square), being the largest, shows compactness at the centre of the distribution, whereas group 39 (red x), being the second largest, is spread across the entire distribution. Group 23 (black square) stands out by being tightly clustered, fully corresponding to all Pinales species (S8 and S9 Figs). Group 3 (teal cross) and 4 (magenta x) are also clearly distinct, both encompassing mostly Myrtales species. Other dimensionality reductions do not show as clear a separation between clusters, but some are reasonably identified regardless of the reduction methodology (S5 and S6 Figs). Note that this visualization is only intended as a qualitative representation of cluster structure.

The majority of the clusters are comprised of species that exhibit functionally unique combinations of traits, often aligning with well known taxonomic structures (e.g., within genus or family). However, we confidently identified one large, taxonomically broad group consisting of 4,291 species with non-distinct or ‘average’ traits, comprising taxa from across many families and orders (Fig 4; see interactive version in S1 Appendix). Other taxonomically dispersed groups, regardless of their size, show low consensus values (S1 Table). Whether this large dispersed group represents ecological generalists, reflects convergent evolution of common trait combinations, or captures species not readily assigned to distinct clusters is a compelling question for future work [81–84].

### Group trait patterns

**Fig 5:**
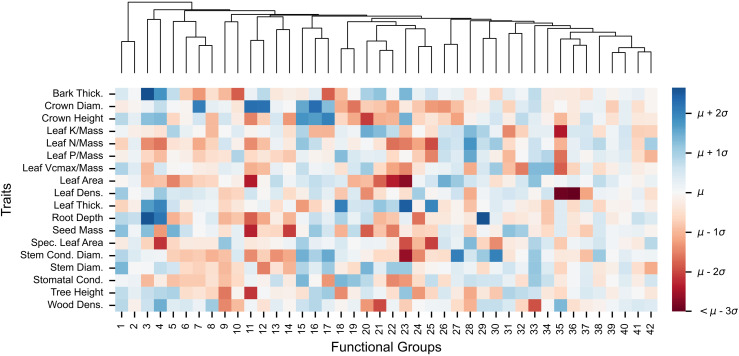
Centroids of 42 functional groups. Mean values were calculated for each group across their species after normalizing the original dataset for each trait. The colour gradient represents these normalized values and is consistent across all traits, with values clipped at 3 standard deviations from the mean to enhance visualization contrast. A dendrogram, constructed using the Ward-linkage method, is included to illustrate the relative consensus distances among the groups.

When exploring the trait patterns underpinning the groups, we see that they are not determined from just one or two traits, but rather all traits appear to be relevant in the identification of different functional groups (Fig 5, S2 Table). The lowest standard deviation across groups is for stem diameter at 70% of the original variation, while the highest is for leaf density at 99%. If a trait was not relevant, we would expect that there is no pattern to the assignment across that particular trait, meaning the standard deviation across groups would be much closer to 0. These results reinforce the idea that group 2 acts as a generalist group, with average values across all traits and low within-group variation, while groups 38–42 also show average trait values, but with much higher variation across species (S7 Fig).

### Functional groups to quantify functional diversity

The value of a functional group approach lies in the fact that functional groups provide a natural analogue of species, allowing the entire suite of species-based diversity metrics to be applied to functional groups. At the simplest level, functional group richness (the number of unique groups in a community) provides an intuitive metric for quantifying functional diversity that doesn’t require complex metrics or calculations. On the other hand, Simpson’s Index—a widely used measure of species-based diversity—can likewise be applied to functional groups, in which case it provides a direct measure of ‘functional redundancy’, i.e., the probability that any two randomly selected species in a community belong to the same functional group. Indeed, any species-based metric can be applied directly to functional group richness, providing relatively simple and intuitive measures of richness, redundancy, divergence, and evenness.

**Fig 6:**
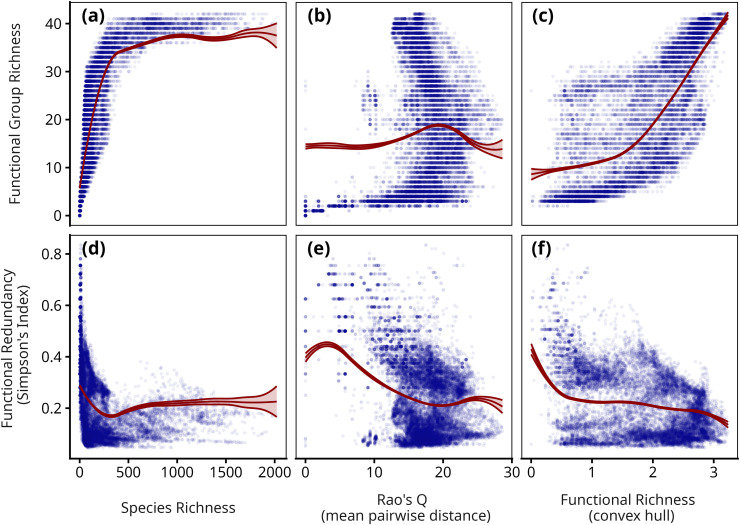
Functional group diversity metrics compared to traditional measures of species richness and functional diversity, calculated for each of *n* = 16,048 forested pixels across the globe (with more than 3 clusters), as reported in Paz et al. [12]. The points show the raw observations, with the lines denoting the fitted trends from generalised additive models, adjusted for spatial autocorrelation (see Methods), along with 95% confidence intervals. **(a-c)** Functional group richness (the number of unique groups) is highly correlated with species richness at low levels, but saturates after approximately 500 species in the system. Functional redundancy is weakly correlated with Rao’s Q, which reflects spatial clustering in trait space, but, compared to functional redundancy exhibits a stronger (albeit still relatively weak) correlation with functional richness. **(d-f)** Functional redundancy (Simpson’s Index applied to groups) is inversely correlated with species richness, revealing significant variation in redundancy at high levels of richness, but is only weakly correlated with Rao’s Q and Functional richness, capturing complementary aspects of both. These results illustrate how traditional diversity metrics can be applied to functional groups, yielding intuitive and simple metrics for quantifying functional composition.

To illustrate how such metrics can be applied to functional groups, we use a recent global dataset of tree community composition [12], which contains presence-absence of around 37,000 tree species at 200-km resolution across the globe. We used our tree-species functional group classification to assign each tree species to a functional group. We then calculated functional redundancy (‘FRedund’, Simpson’s Index) and functional group richness (‘FGR’, number of groups) across all species contained within each pixel. We then compared these group-derived metrics with three traditional diversity metrics reported in Paz et al.: species richness (number of unique species), mean pairwise distance (Rao’s quadratic entropy, a measure of functional clustering), and functional richness (convex hull volume across all traits) (see Fig 6).

These results demonstrate how functional group diversity complements and reflects key elements of traditional metrics, while also providing novel insight into the functional structure of natural communities. After accounting for spatial autocorrelation, Functional Group Richness (FGR) exhibits a saturating positive relationship with traditional species richness (Pearson *r* = 0.58, p < 0.001; Fig 6a), which flattens after ∼500 species, and likewise shows a strong linear correlation with functional richness (*r* = 0.74, p < 0.001). Conversely, FGR has weak residual correlation with Rao’s Q (*r* = 0.15, p < 0.001; Fig 6b), highlighting that this metric is largely independent of distance-based similarity. Functional Redundancy, on the other hand, aims to capture overlap between species, and accordingly exhibits a moderate negative correlation with Rao’s Q (*r* = -0.40, p < 0.001; Fig 6e), along with a negligible correlation with species richness (*r* = -0.04, p < 0.001) and a slight negative relationship with functional richness (*r* = -0.21 p < 0.001; Fig 6f). Collectively, these results illustrate how group-based diversity metrics can provide complementary insight into functional structure by integrating different aspects of species richness, functional clustering, and functional breadth (also see S15 Fig for spatial projections). Moreover, the simplicity of group-based calculations helps to overcome some of the computational limitations of existing functional diversity metrics (e.g., the inability to calculate convex hull volume in communities where there are fewer species than traits) while also being based on well-known, interpretable species-diversity metrics.

## Discussion

Here, we present a clustering framework that enables the identification of functional groups while explicitly accounting for trait uncertainty and trait correlation. By generating multiple scenarios of a trait dataset and clustering each independently, we capture the variability inherent in imputed trait values or in the presence of intraspecific variation. By aggregating these results into a consensus matrix and applying hierarchical clustering, we are able to identify robust functional groups and their corresponding level of certainty (consensus). In doing this, this approach increases agreement across bootstrap samples and reduces variation relative to a single clustering approach (S4 Fig). When applied to a global dataset of tree species, this approach identified 42 functional groups, most of which exhibited moderate to high consensus. We also show how these groups can be interpreted in terms of their average trait composition and linked to other biodiversity metrics across space.

In terms of our methodology, our main novelty relied on the use of consensus clustering with resamples of the prediction error and the optimization of groups in each step of consensus clustering. Consensus clustering has been used before, but mainly to reach consensus between different clustering methods, or by using bootstrapping in the original dataset [49,85]. To our knowledge, these techniques have not been used to solve the problem of trait uncertainty. Other methods have been proposed to solve this issue, like incorporating trait uncertainty into a Mixture Model [86–88], but the complexity quickly scales, and it becomes practically infeasible when we also have uncertainty in the number of groups. Our model provides a novel, scalable solution to addressing the issues of trait uncertainty and trait collinearity, especially suited for datasets constructed using imputed traits, as is the case in nearly all large trait databases.

In the case study, our consensus clustering approach resulted in the identification of 42 functional groups of tree species worldwide, of which 36 demonstrated an average consensus greater than 20%, indicating a good level of agreement, and 9 of these groups had a consensus exceeding 50%, reflecting high agreement. The groups with high consensus exhibited complex trait patterns, suggesting that all traits play a relevant role at some partition level. Conversely, the 6 groups with less than 20% consensus underscore the significance of accounting for uncertainty and highlight the need for further data collection. Our findings also reveal that while functional groups share some overlap with taxonomic groups, the observed differences in phylogenetic distance and consensus underscore the importance of distinguishing between taxonomic and functional classifications. While we only consider global trait patterns, a clear extension is to rerun these methods on local scales to identify functional (sub)groups within specific regions. For example, using only species from tropical rainforests or boreal forests could reveal detailed functional patterns, overlooked at a global scale, due to the distinct structures of resource competition in each region [9,66,89,90].

The key benefit of a functional group approach is that it allows us to directly apply biodiversity metrics constructed for taxonomic data, such as species richness, Simpson index, and Shannon-Wiener index [36,37,91]. In addition, the complete consensus matrix allows us to apply continuous diversity metrics designed for phylogenetic-like dendrograms, such as Faith’s phylogenetic diversity [7]. In doing this, the method and framework proposed here are intended to overcome some of the challenges in adopting functional diversity metrics in conservation and restoration, by providing simple and intuitive metrics that do not rely on having access to the original trait database or expert knowledge of the dozens of different functional diversity metrics. As a simple example, when doing species selection for reforestation, practitioners could directly use the list of tree functional groups to select focal species to ensure that their target composition contains a diversity of functional roles and a low level of functional redundancy. Similarly, current efforts to quantify and track biodiversity are largely limited to measures of species richness, but these species-based metrics can easily be converted into functional diversity metrics by applying functional group labels to common diversity calculations. Because a list of functional groups is simple to share and access, such approaches are repeatable and transparent, facilitating adoption and comparison of results across taxa and study sites.

An important distinction between our approach and traditional functional group classification is that our method is unsupervised, as opposed to the original approach of assigning groups based on expert knowledge [20]. Thus, there is no ground truth for the resulting group assignments, and while this method provides a data-driven approach that minimizes human bias, it also means that the resulting clusters may not reflect the ecological question of interest. Indeed, we used Ward’s linkage method in our case study to identify compact spherical clusters, in line with expectations from niche theory [58,33]; other linkage methods that likewise favor compact groups, such as average linkage, would be expected to yield similar results to Ward’s (S13 Fig). However, alternate approaches may work best in other ecological settings. For example, if one expects ecologically relevant clusters to track environmental gradients, then single linkage might be better suited due to its ability to chain together clusters [92].

Similarly, we use BIC-based cluster-size selection when a likelihood is available (e.g., for individual cluster selection across the resamples, Fig 1a), and otherwise we use silhouette scores in the absence of a likelihood (e.g., for the consensus matrix, Fig 1c). In practice, any cluster-size selection method is valid, and these criteria can and will yield different results and cluster assignments, such that the user is encouraged to compare approaches and tailor these decisions to their application and question of interest. For example, here, by comparing the functional groups to underlying phylogenetic structure or trait distribution (e.g., Fig 5), we show how the resulting groups can be linked to known ecological roles and species labels, facilitating group interpretation and aiding in downstream analysis. Future steps to validate these groups could include incorporating known categories, such as the presence of species in different biomes [93,94], functional roles labelled by experts [69,95], temperature and precipitation niches [96,97], and environmental tolerances [83,98].

As with any unsupervised clustering method, the choice of clustering algorithm is an important decision. For building a clustering ensemble, we used Gaussian mixture models for their ability to handle multivariate trait correlation, which makes them less sensitive to trait collinearity or trait omission. However, such models still assume a specific cluster distribution that can be hard to validate in practice [53]. An alternative approach would be a non-parametric, density-based method like HDBSCAN [99], which may perform better when functional groups are complex and non-spherical, but its sensitivity to observation distance can lead to unstable clustering results (S11 and S12 Figs). Furthermore, it is not clear that a density-based cluster would reflect what we understand as a functional group, since these methods allow for species very far apart in functional space to be classified as the same group if they are connected by other species (e.g., a horseshoe-shaped pattern where all species are in the same group). Thus, future work could explore density-based perturbations to improve HDBSCAN’s stability [100], and comparison of such approaches to distance-based methods, which could aid in the identification of novel groups and ecological similarities across species. On the other hand, a key benefit of the hierarchical consensus clustering approach used here is that it naturally allows for further subdivisions, such that these groups could be further partitioned into smaller subgroups and compared to deeper taxonomic levels like family and genus. The method presented here is thus intended as a general and flexible framework for assigning functional groups, which can be tailored to the specific question of interest.

## Conclusion

Collectively, our study presents a novel framework for functional group classification that addresses trait uncertainty and trait correlation. By applying this method to a case study of global tree traits, we show how this approach can be used to identify ecologically meaningful clusters and the traits underpinning cluster assignment. By applying traditional species-based diversity metrics to functional groups, we further illustrate how functional groups can be used to generate interpretable, intuitive functional diversity metrics that allow for scalable and repeatable comparisons across study systems. In doing so, this approach provides a scalable method for incorporating functional biodiversity into restoration or conservation activities, and for quantifying and tracking changes in biodiversity through time.

## Supporting information

S1 Table Detail of group size and group average consensus for each of the functional groups as displayed in Fig 3. (PDF)

S2 Table Detailed trait statistics for cluster centroids after standardizing each trait as shown in Fig 5. Mean values near 0 and standard deviation near 1 indicates that it recreates the original distribution.
(PDF)

S1 Appendix Interactive t-SNE. Interactive version of Fig 4 with trait axes and species names. Trait loadings on each axis were calculated using multivariate regression, with the figure displaying only those traits with partial correlation *r* > 0.3.
(HTML)

S1 Fig Correlation matrix for all 18 traits. Correlation matrix for all 18 traits (after natural log), with a color scale where blue denotes positive correlations and red signifies negative correlations. Most traits exhibit significant correlations, highlighting the necessity of accounting for these relationships in clustering and distance analyses.
(PDF)

S2 Fig Trait distribution across the dataset. Trait distribution for the complete dataset of 18 traits (after natural log transformation), showing spikes due to repeated imputation. The blend of continuous and discrete distributions underscores the issue of trait uncertainty in clustering algorithms.
(PDF)

S3 Fig Error distributions for traits. Distribution of errors across traits for angiosperms (a) and gymnosperms (b). Errors are calculated by subtracting log predicted values from log observed values for species with available data. The x-axis represents logged trait error values, and the y-axis represents frequency.
(PNG)

S4 Fig Adjusted Rand Index Distribution. We evaluate clustering consistency on a subsample of 1,365 species (including all gymnosperms to ensure structural heterogeneity) using two approaches. In the first approach (blue), a Gaussian Mixture Model (GMM) is applied independently to each of 500 resampled datasets, producing 500 clustering results. The Adjusted Rand Index (ARI) is then computed for all pairwise comparisons among these clusterings, reflecting the variability of the method across resamples. In the second approach (orange), the 500 resampled clusterings are partitioned into 10 groups of 50. Within each group, a consensus clustering is derived using the full consensus framework, yielding 10 consensus clusterings. The ARI is then computed for all pairwise comparisons among these consensus results, providing a measure of agreement after aggregation.
(PNG)

S5 Fig PCA clustering results. Functional groups identified through Principal Component Analysis (PCA) for dimensionality reduction. PCA transforms the data into principal components that capture the most variance, revealing considerable group overlap and clustering, particularly away from the center of the distribution.
(PNG)

S6 Fig UMAP clustering results. Functional groups identified using Uniform Manifold Approximation and Projection (UMAP) for dimensionality reduction. UMAP reduces data complexity by preserving local and global structures, with groups outside the distribution center showing greater dispersion.
(PNG)

S7 Fig Group-level standard deviation of traits. Standard deviation of log-standardized trait values within each functional group. Higher values indicate greater within-group variation.
(PDF)

S8 Fig Taxonomic group composition by functional groups. Each taxonomic group composition in terms of functional groups, with proportions displayed for each taxonomic group. Rows sum to 1.
(PDF)

S9 Fig Functional group composition by taxonomic groups. Composition of functional groups by taxonomic groups, showing the proportion of each functional group belonging to specific taxonomic groups. Rows sum to 1.
(PDF)

S10 Fig GMM results without resampling. Visualization of clusters in t-SNE space, where the central region suggests overfitting due to repeated observations. The optimal BIC is observed at 800 components.
(PNG)

S11 Fig HDBSCAN results on original data. Clustering using HDBSCAN with a minimum cluster size of 10 species per group. Black circles indicate noise points. Overfitting issues due to imputed values are evident.
(PNG)

S12 Fig HDBSCAN results for consensus clustering. t-SNE visualization for HDBSCAN clusters, showing two main clusters and challenges in identifying smaller groups.
(PNG)

S13 Fig Average linkage clustering results. The t-SNE visualization obtained using the average linkage method yields 34 clusters, compared to 42 clusters produced by the Ward method. This discrepancy highlights the sensitivity of clustering outcomes to the choice of linkage criterion, which should be selected in accordance with the underlying assumptions about cluster structure. Notably, the adjusted Rand index between the two clustering solutions is 0.51, indicating a moderate level of agreement and a substantial overlap in the resulting partitions.
(PNG)

S14 Fig Species-level stability. Histogram of species stability values computed from the consensus matrix, where stability is defined as the ratio between the average similarity of a species to members of its assigned cluster and its average similarity to all species. Higher values indicate more consistent co-clustering across resamples.
(PNG)

S15 Fig Global projections of functional group metrics. (a) Functional Richness is the number of functional groups per pixel (200 km nominal diameter hexagonal bins), and (b) Functional Redundancy is Simpson’s Index applied to the functional groups, here shown on the log scale for better spatial resolution. Compare to the results of Paz et al. [12].
(PNG)
