# Supplementary material for: Functional group classification using consensus clustering
Source: PLoS Comput Biol. 2026 May 13;22(5):e1014278. doi: 10.1371/journal.pcbi.1014278 (PMC13197079; doi:10.1371/journal.pcbi.1014278)
Supplement: S1 Table — (PDF) [file pcbi.1014278.s001.pdf]

Group number	Number of species	Average consensus
1	613	0.36
2	4,291	0.23
3	950	0.67
4	1,058	0.70
5	1,144	0.43
6	1,703	0.22
7	601	0.44
8	1,471	0.29
9	1,188	0.43
10	1,584	0.35
11	366	0.68
12	1,159	0.49
13	1,452	0.31
14	1,107	0.41
15	856	0.47
16	1,355	0.34
17	690	0.42
18	324	0.57
19	1,082	0.39
20	717	0.57
21	460	0.54
22	361	0.44
23	561	0.83
24	448	0.37
25	814	0.40
26	1,326	0.38
27	738	0.32
28	1,297	0.37
29	663	0.55
30	755	0.49
31	1,315	0.31
32	1,299	0.31
33	1,079	0.39
34	1,958	0.20
35	384	0.69
36	541	0.39
37	1,257	0.15
38	1,815	0.20
39	3,994	0.07
40	1,387	0.07
41	1,040	0.14
42	625	0.19
