# Supplementary material for: Functional group classification using consensus clustering
Source: PLoS Comput Biol. 2026 May 13;22(5):e1014278. doi: 10.1371/journal.pcbi.1014278 (PMC13197079; doi:10.1371/journal.pcbi.1014278)
Supplement: S2 Table — Mean values near 0 and standard deviation near 1 indicates that it recreates the original distribution. (PDF) [file pcbi.1014278.s002.pdf]

	Mean	Std	Max		Min	
			Value	Group	Value	Group
Bark Thick.	0.05	0.84	2.64	3	-1.82	10
Crown Diam.	0.03	0.97	2.29	16	-1.60	19
Crown Height	-0.05	0.94	2.02	17	-2.08	20
Leaf K/Mass	0.01	0.78	1.48	20	-2.40	35
Leaf N/Mass	-0.15	0.89	1.76	28	-1.94	25
Leaf P/Mass	-0.10	0.68	1.52	28	-1.64	35
Leaf Vcmax/Mass	-0.09	0.76	1.32	33	-1.78	35
Leaf Area	-0.16	0.95	1.46	26	-2.69	23
Leaf Dens.	-0.11	0.99	1.12	4	-4.19	36
Leaf Thick.	0.16	0.84	2.46	23	-1.27	15
Root Depth	0.00	0.95	2.60	29	-1.77	11
Seed Mass	-0.13	0.89	1.42	5	-2.31	11
Spec. Leaf Area	-0.12	0.78	1.31	28	-2.16	4
Stem Cond. Diam.	-0.05	0.98	2.03	27	-2.61	23
Stem Diam.	0.06	0.70	1.39	20	-1.51	12
Stomatal Cond.	-0.02	0.73	1.38	18	-1.44	22
Tree Height	-0.08	0.76	1.17	23	-2.14	11
Wood Dens.	0.00	0.78	1.46	4	-1.97	21
