# Supplementary material for: Functional group classification using consensus clustering
Source: PLoS Comput Biol. 2026 May 13;22(5):e1014278. doi: 10.1371/journal.pcbi.1014278 (PMC13197079; doi:10.1371/journal.pcbi.1014278)
Supplement: S2 Fig — Trait distribution for the complete dataset of 18 traits (after natural log transformation), showing spikes due to repeated imputation. The blend of continuous and discrete distributions underscores the issue of trait uncertainty in clustering algorithms. (PDF) [file pcbi.1014278.s005.pdf]

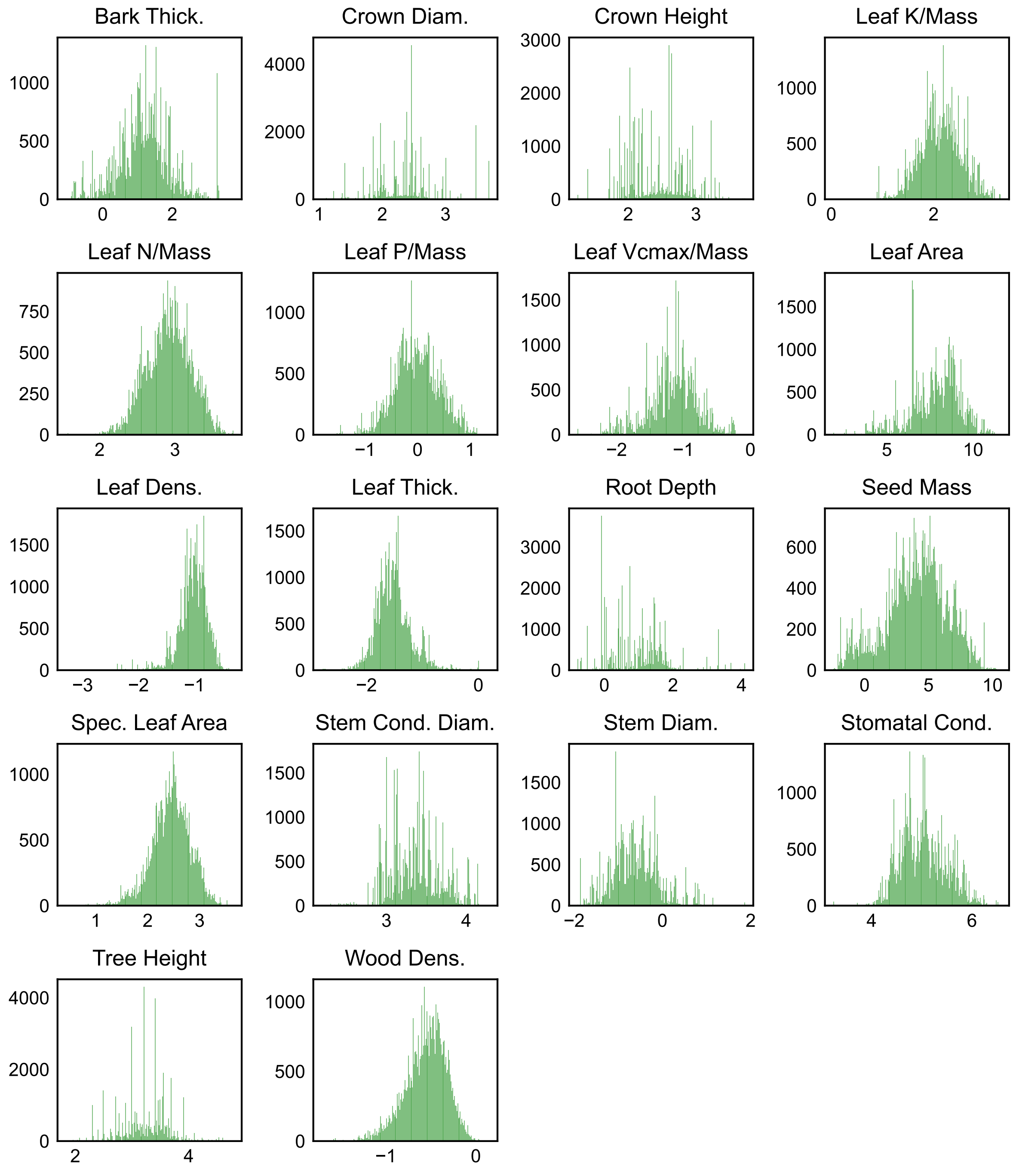
Bark Thick.
0 500 1000
0 2
Crown Diam.
0 2000 4000
1 2 3
Crown Height
0 1000 2000 3000
2 3
Leaf K/Mass
0 500 1000
0 2
Leaf N/Mass
0 250 500 750
2 3
Leaf P/Mass
0 500 1000
−1 0 1
Leaf Vcmax/Mass
0 500 1000 1500
−2 −1 0
Leaf Area
0 500 1000 1500
5 10
Leaf Dens.
0 500 1000 1500
−3 −2 −1
Leaf Thick.
0 500 1000 1500
−2 0
Root Depth
0 1000 2000 3000
0 2 4
Seed Mass
0 200 400 600
0 5 10
Spec. Leaf Area
0 500 1000
1 2 3
Stem Cond. Diam.
0 500 1000 1500
3 4
Stem Diam.
0 500 1000 1500
−2 0 2
Stomatal Cond.
0 500 1000
4 6
Tree Height
0 2000 4000
2 4
Wood Dens.
0 500 1000
−1 0
