# Supplementary figures and images for: Functional group classification using consensus clustering
Source: PLoS Comput Biol. 2026 May 13;22(5):e1014278. doi: 10.1371/journal.pcbi.1014278 (PMC13197079; doi:10.1371/journal.pcbi.1014278)

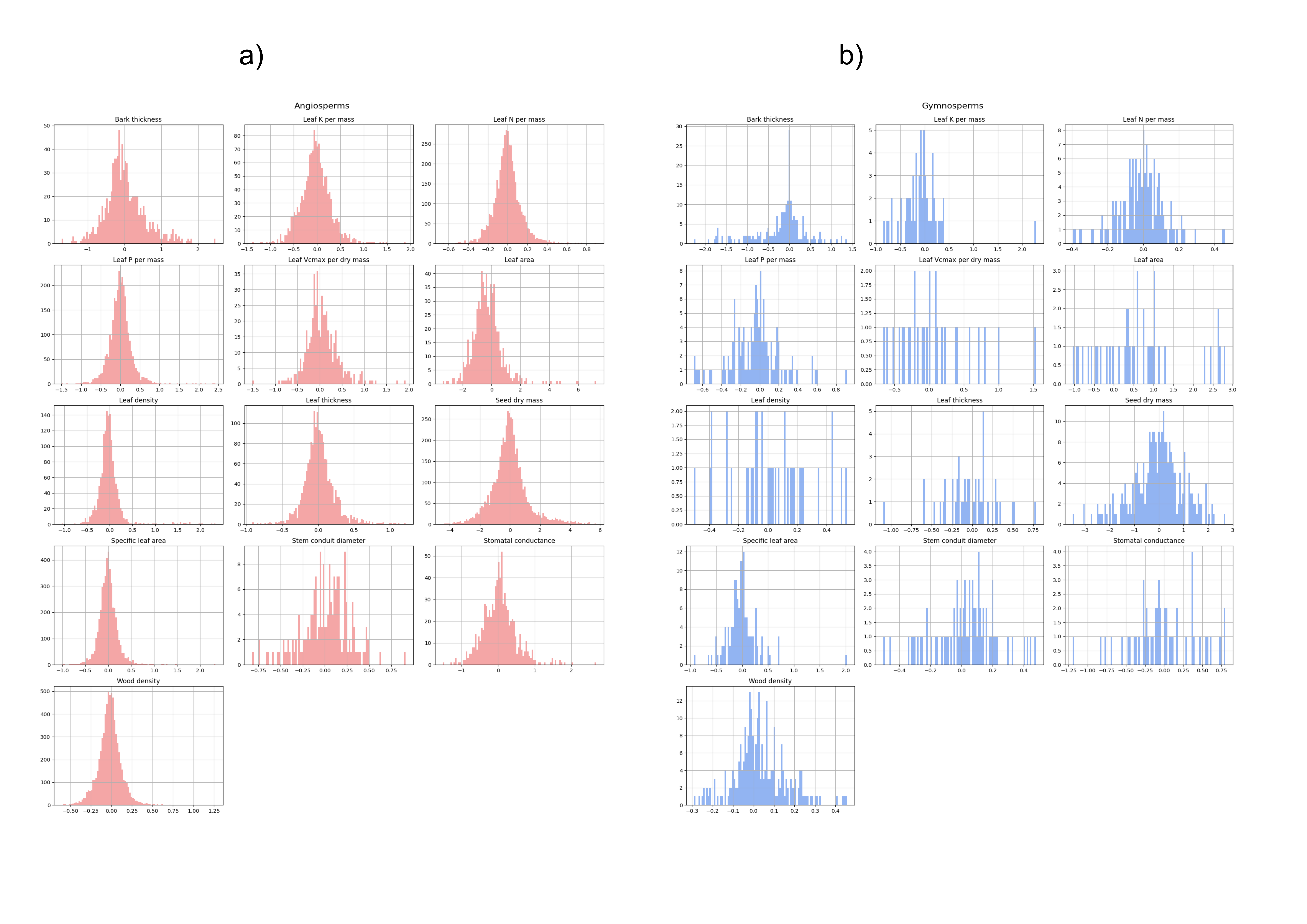

Supplement: S3 Fig — Distribution of errors across traits for angiosperms (a) and gymnosperms (b). Errors are calculated by subtracting log predicted values from log observed values for species with available data. The x-axis represents logged trait error values, and the y-axis represents frequency. (PNG) [file pcbi.1014278.s006.png]

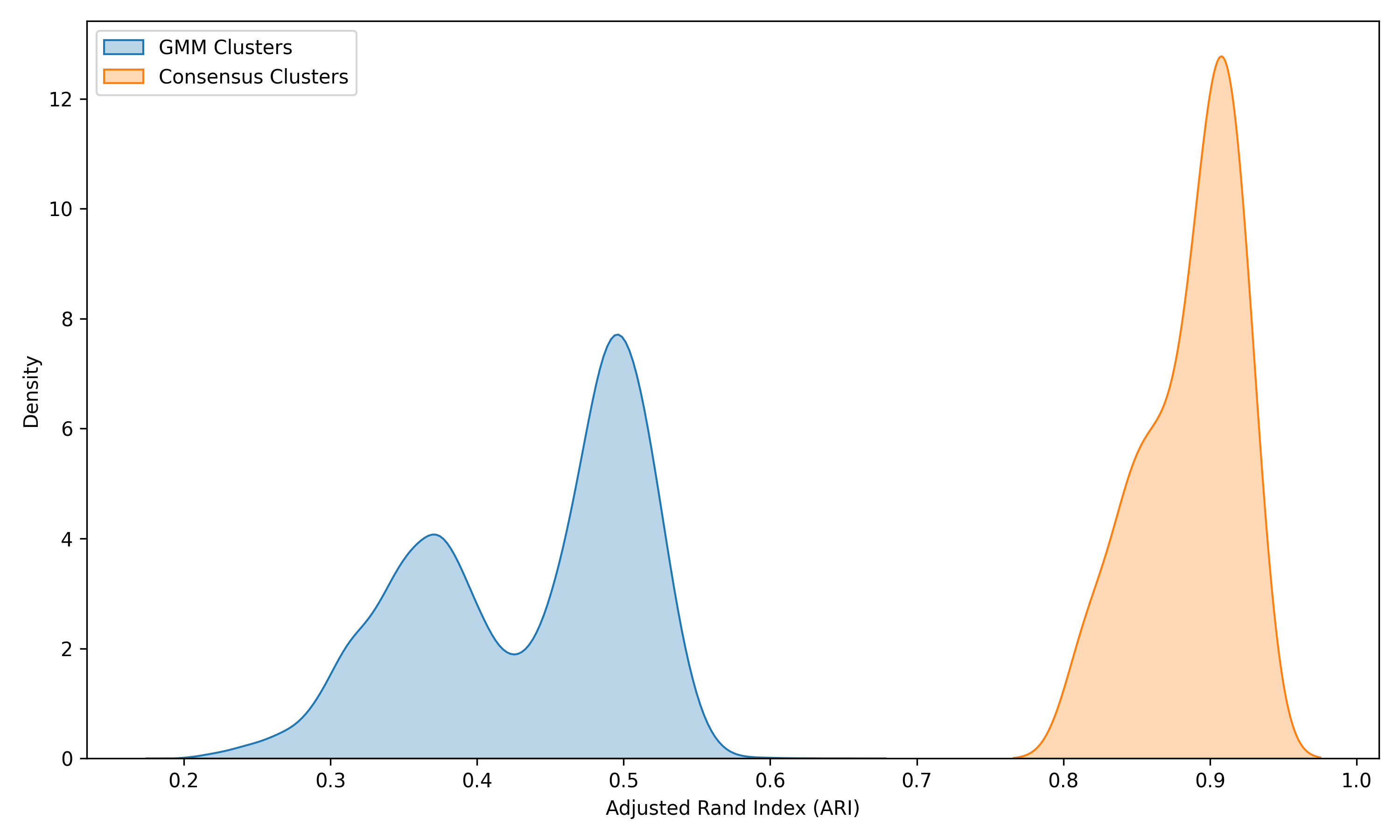

Supplement: S4 Fig — We evaluate clustering consistency on a subsample of 1,365 species (including all gymnosperms to ensure structural heterogeneity) using two approaches. In the first approach (blue), a Gaussian Mixture Model (GMM) is applied independently to each of 500 resampled datasets, producing 500 clustering results. The Adjusted Rand Index (ARI) is then computed for all pairwise comparisons among these clusterings, reflecting the variability of the method across resamples. In the second approach (orange), the 500 resampled clusterings are partitioned into 10 groups of 50. Within each group, a consensus clustering is derived using the full consensus framework, yielding 10 consensus clusterings. The ARI is then computed for all pairwise comparisons among these consensus results, providing a measure of agreement after aggregation. (PNG) [file pcbi.1014278.s007.png]

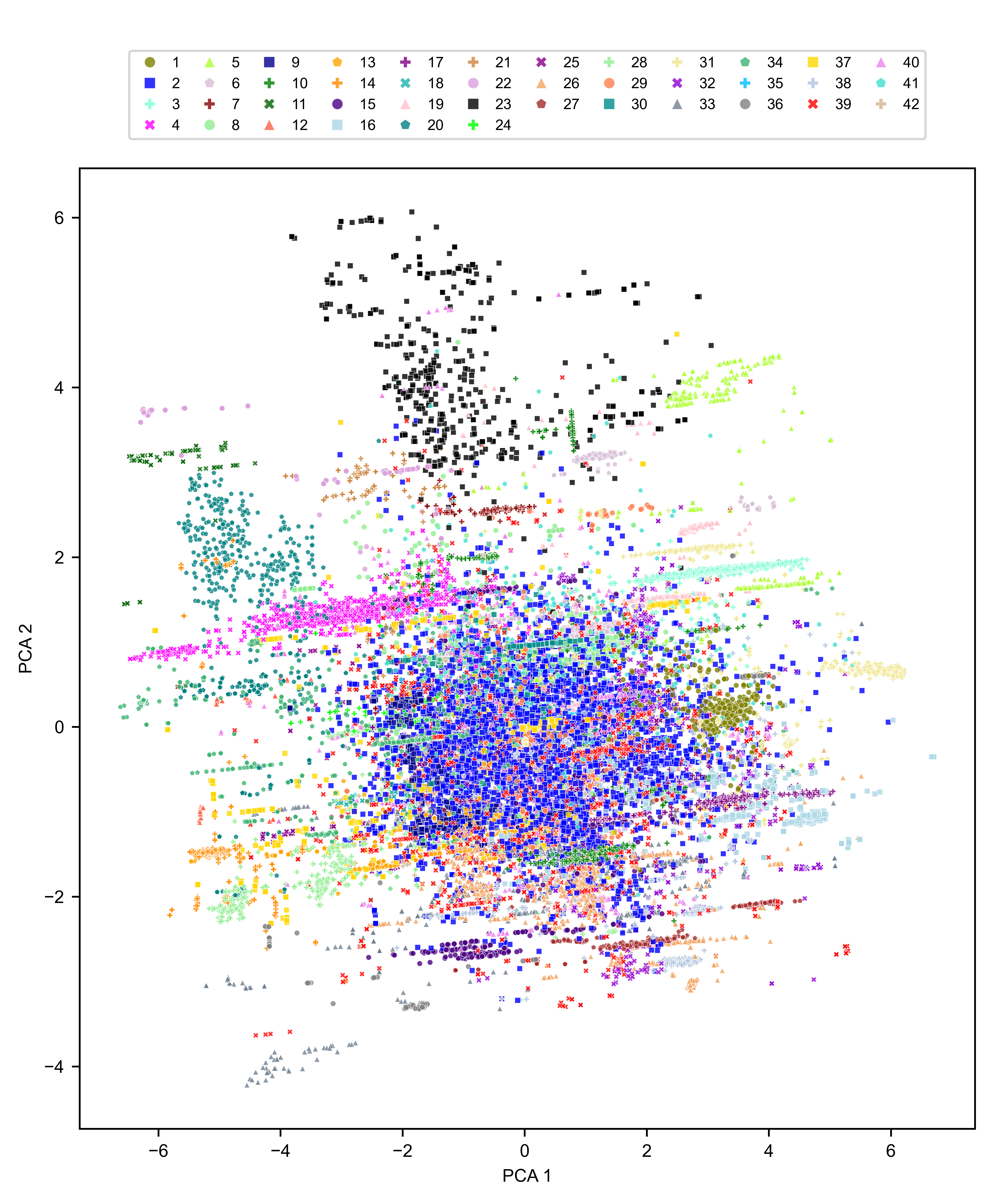

Supplement: S5 Fig — Functional groups identified through Principal Component Analysis (PCA) for dimensionality reduction. PCA transforms the data into principal components that capture the most variance, revealing considerable group overlap and clustering, particularly away from the center of the distribution. (PNG) [file pcbi.1014278.s008.png]

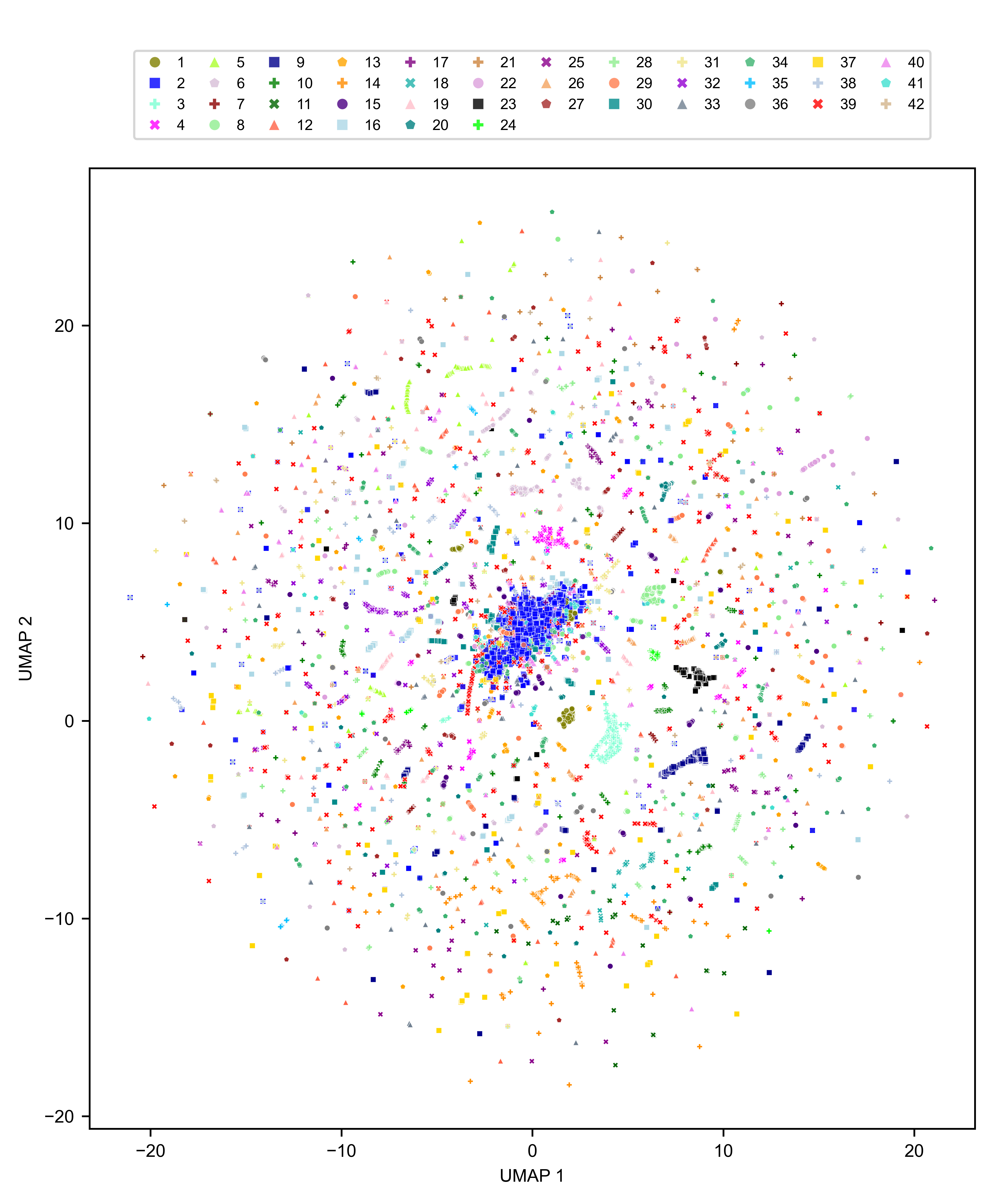

Supplement: S6 Fig — Functional groups identified using Uniform Manifold Approximation and Projection (UMAP) for dimensionality reduction. UMAP reduces data complexity by preserving local and global structures, with groups outside the distribution center showing greater dispersion. (PNG) [file pcbi.1014278.s009.png]

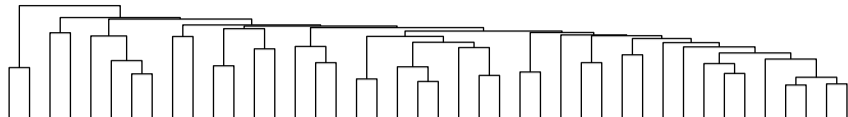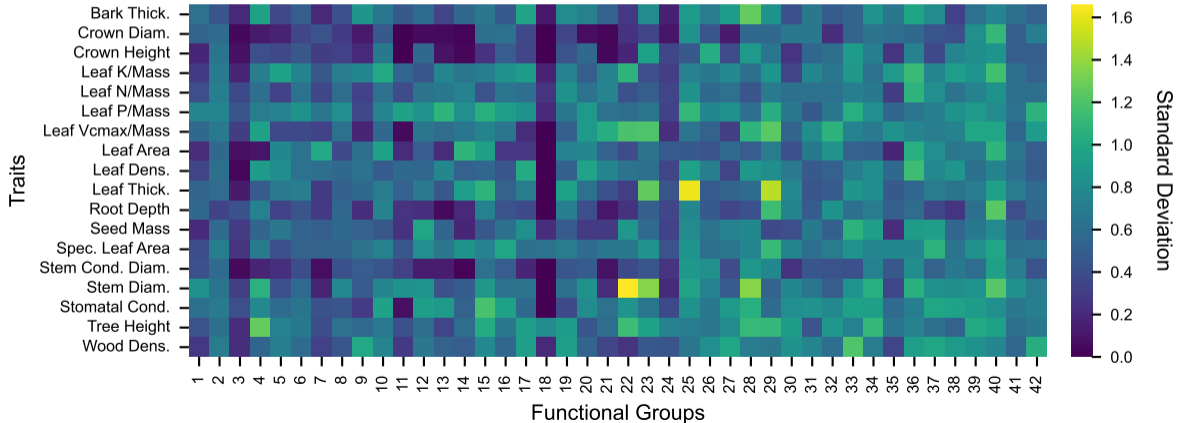

Supplement: S7 Fig — Standard deviation of log-standardized trait values within each functional group. Higher values indicate greater within-group variation. (PDF) [file pcbi.1014278.s010.pdf]

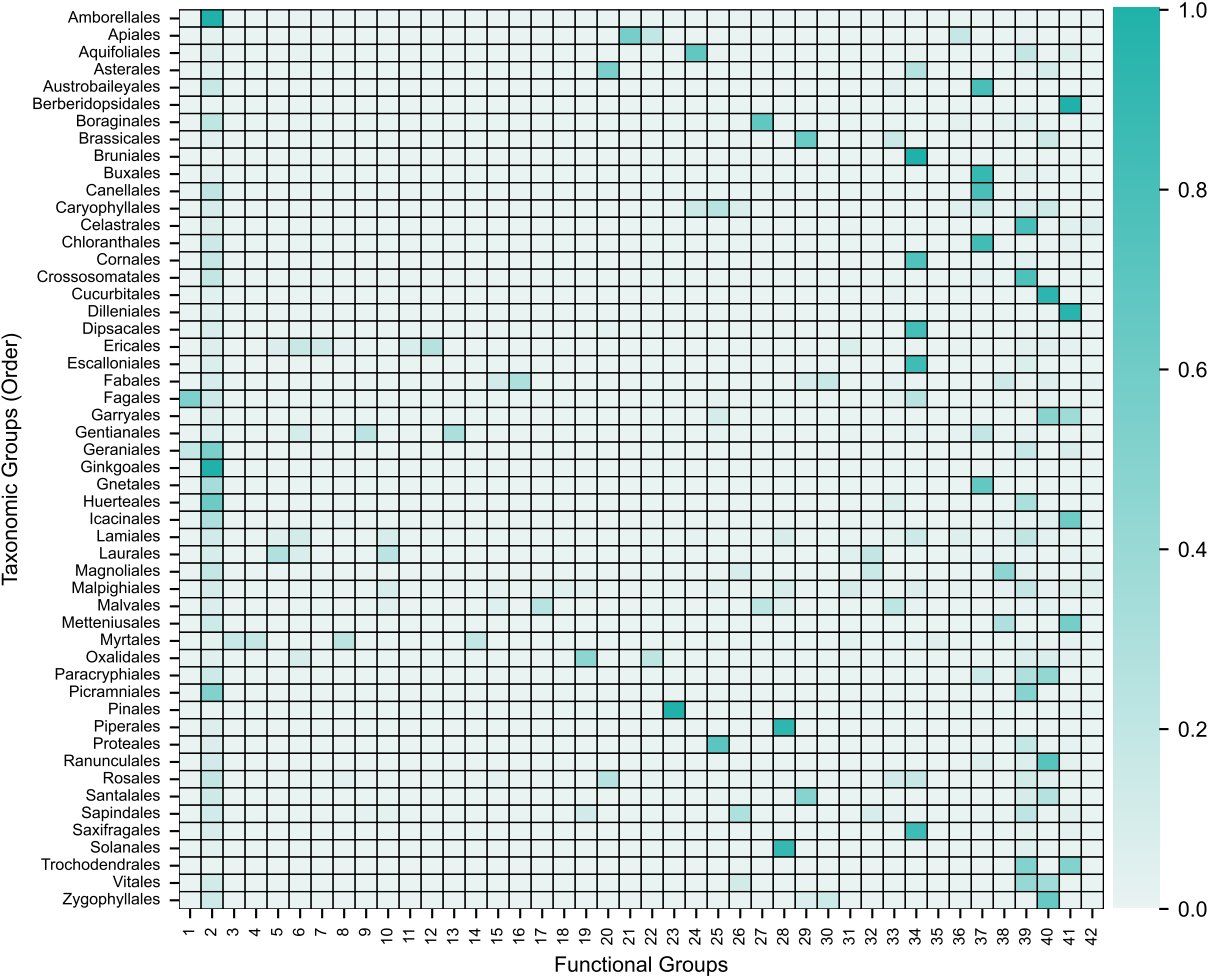

Supplement: S8 Fig — Each taxonomic group composition in terms of functional groups, with proportions displayed for each taxonomic group. Rows sum to 1. (PDF) [file pcbi.1014278.s011.pdf]

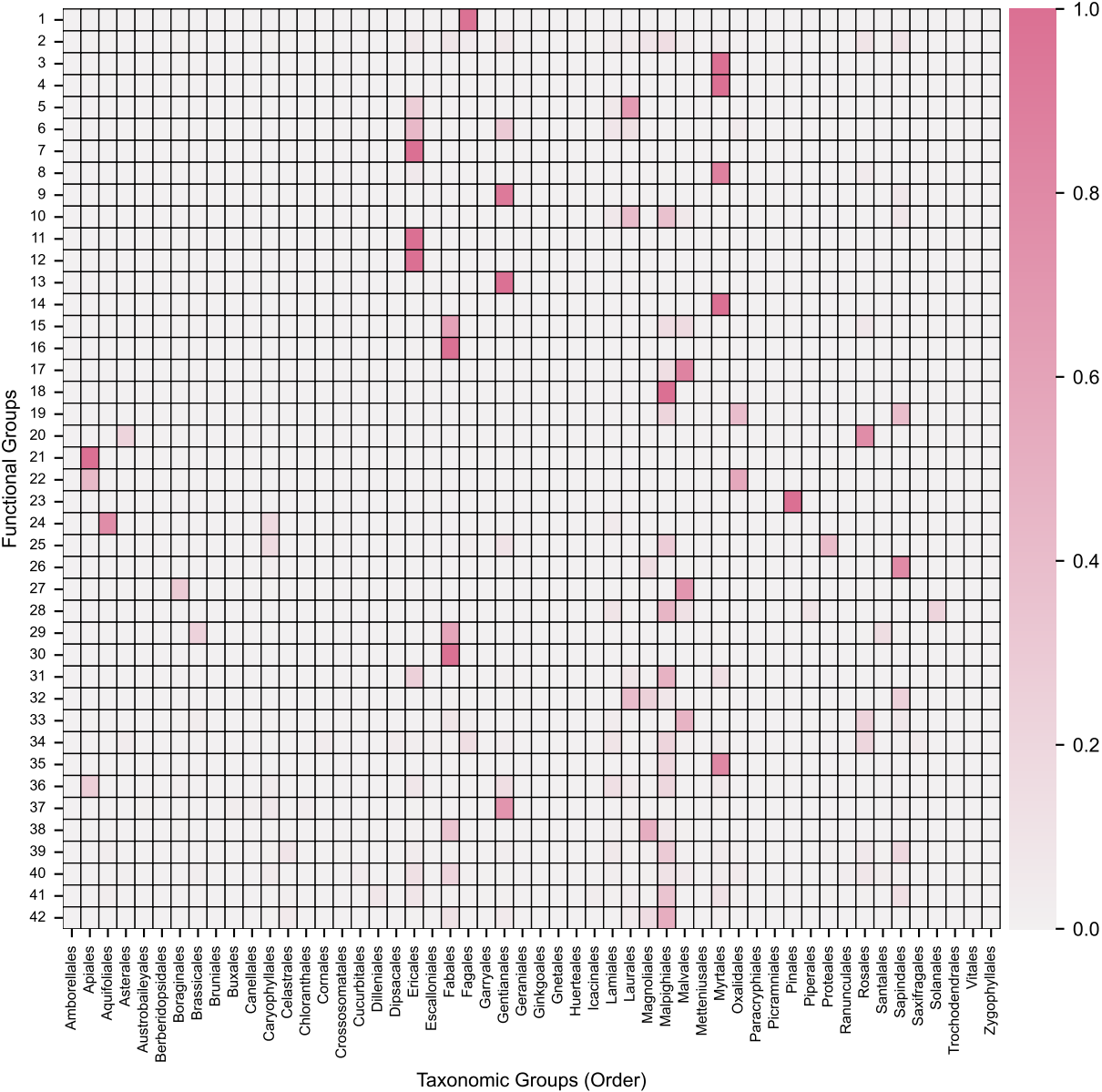

Supplement: S9 Fig — Composition of functional groups by taxonomic groups, showing the proportion of each functional group belonging to specific taxonomic groups. Rows sum to 1. (PDF) [file pcbi.1014278.s012.pdf]

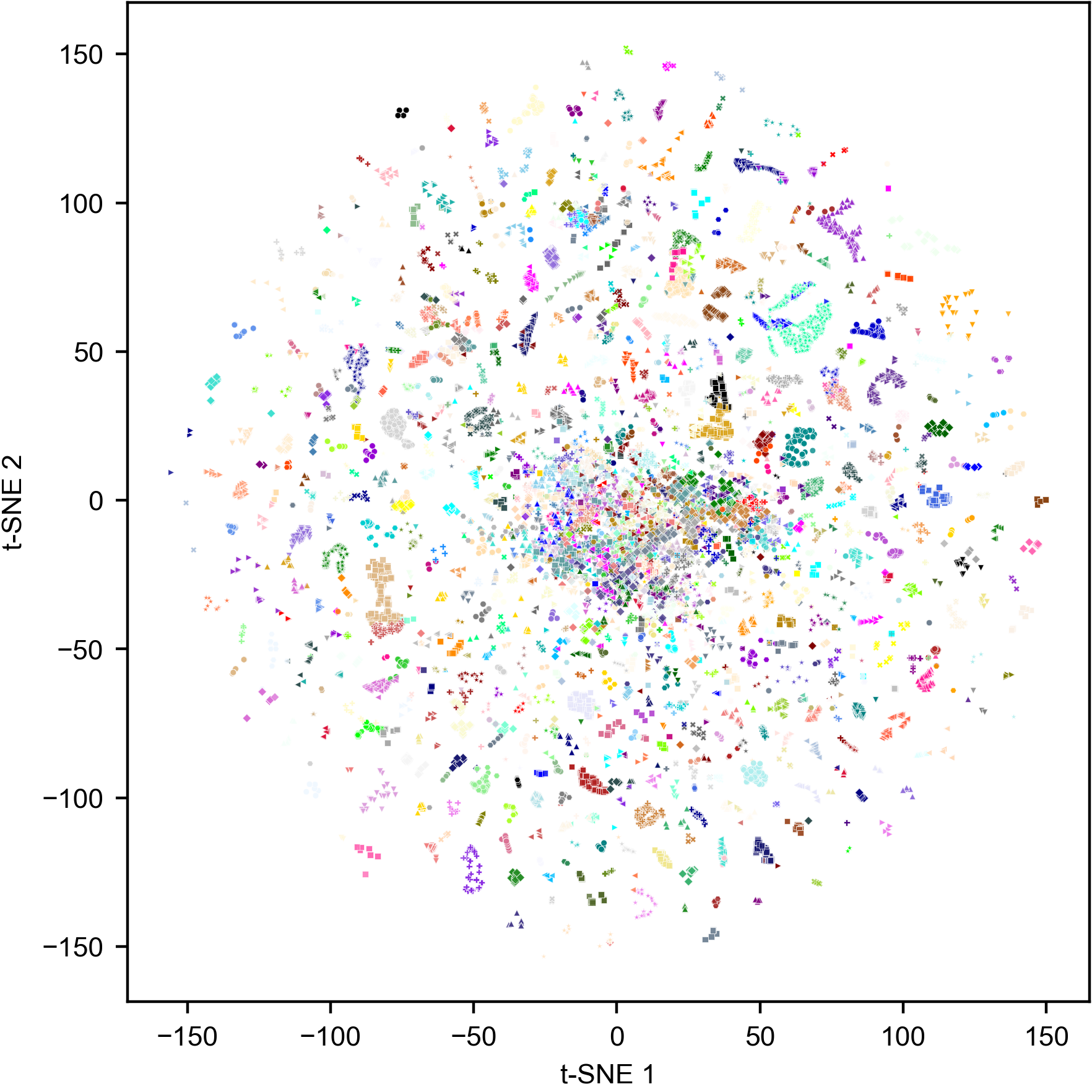

Supplement: S10 Fig — Visualization of clusters in t-SNE space, where the central region suggests overfitting due to repeated observations. The optimal BIC is observed at 800 components. (PNG) [file pcbi.1014278.s013.png]

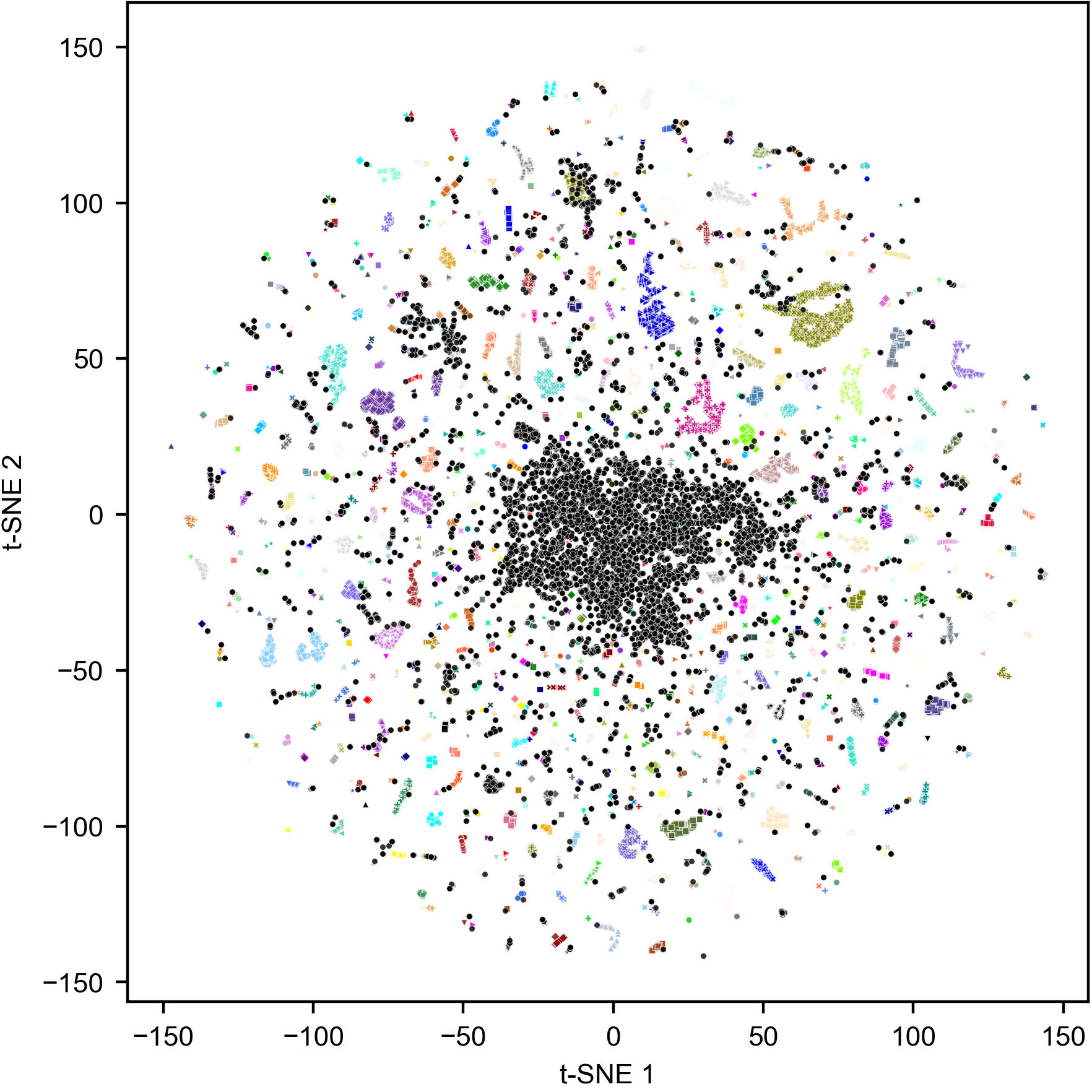

Supplement: S11 Fig — Clustering using HDBSCAN with a minimum cluster size of 10 species per group. Black circles indicate noise points. Overfitting issues due to imputed values are evident. (PNG) [file pcbi.1014278.s014.png]

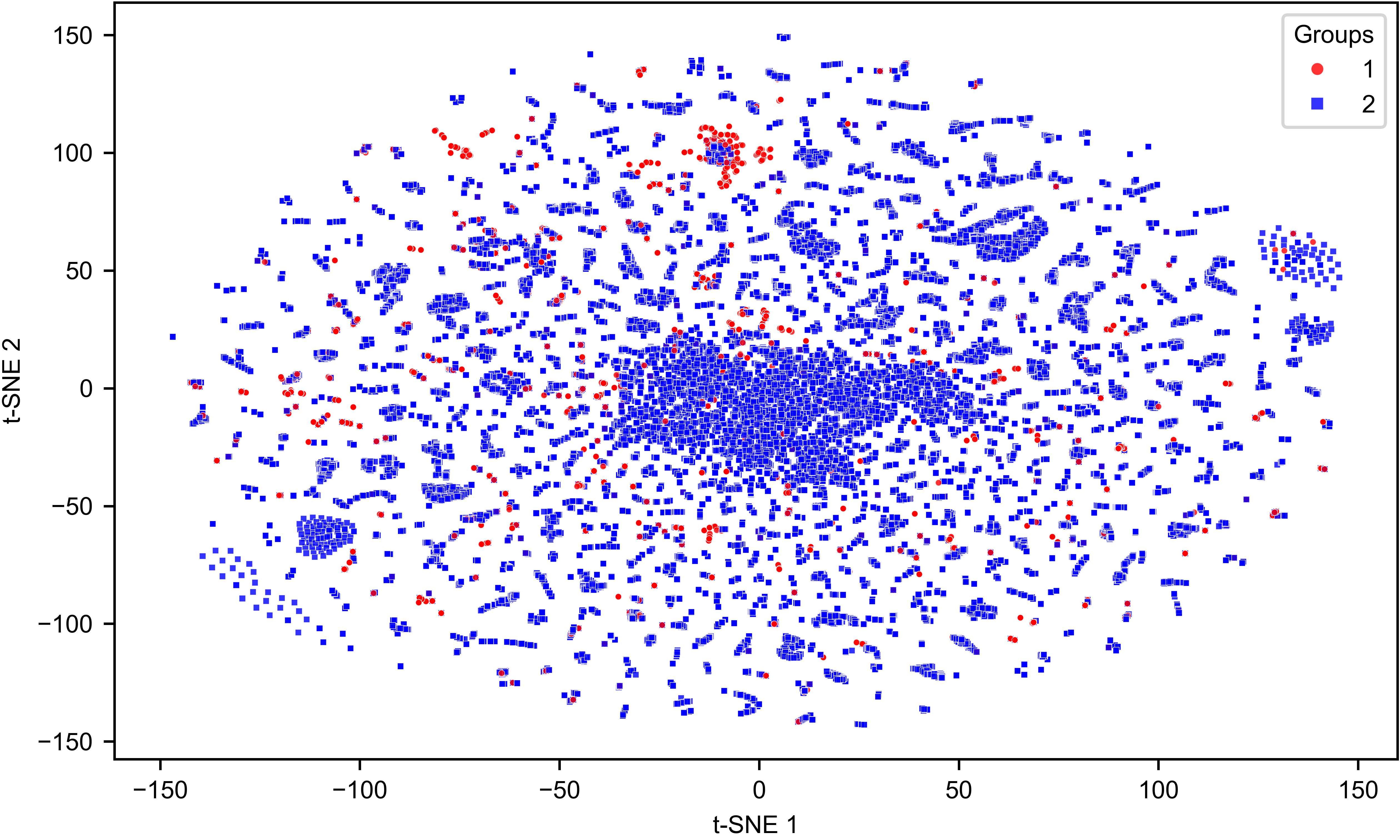

Supplement: S12 Fig — t-SNE visualization for HDBSCAN clusters, showing two main clusters and challenges in identifying smaller groups. (PNG) [file pcbi.1014278.s015.png]

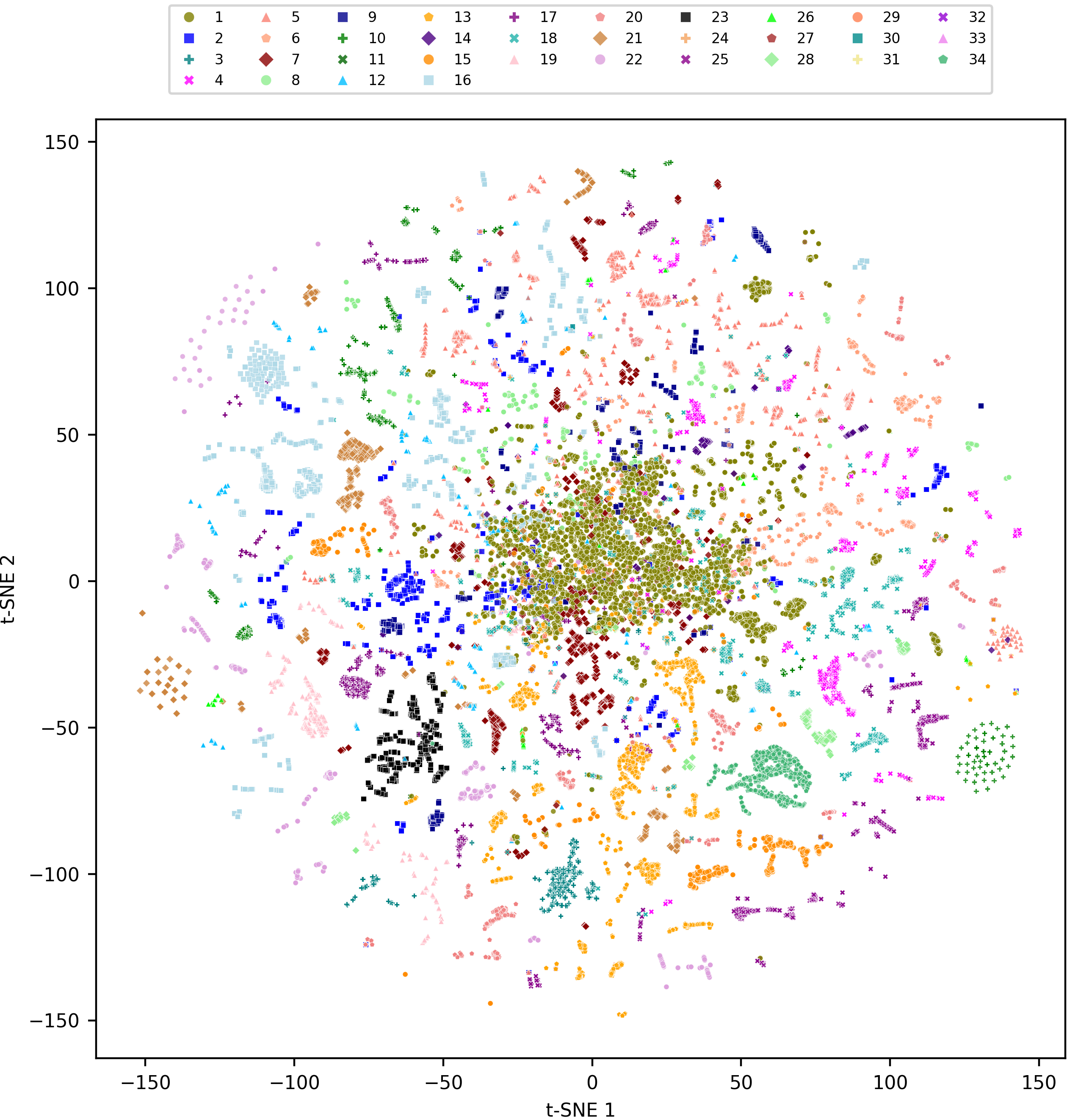

Supplement: S13 Fig — The t-SNE visualization obtained using the average linkage method yields 34 clusters, compared to 42 clusters produced by the Ward method. This discrepancy highlights the sensitivity of clustering outcomes to the choice of linkage criterion, which should be selected in accordance with the underlying assumptions about cluster structure. Notably, the adjusted Rand index between the two clustering solutions is 0.51, indicating a moderate level of agreement and a substantial overlap in the resulting partitions. (PNG) [file pcbi.1014278.s016.png]

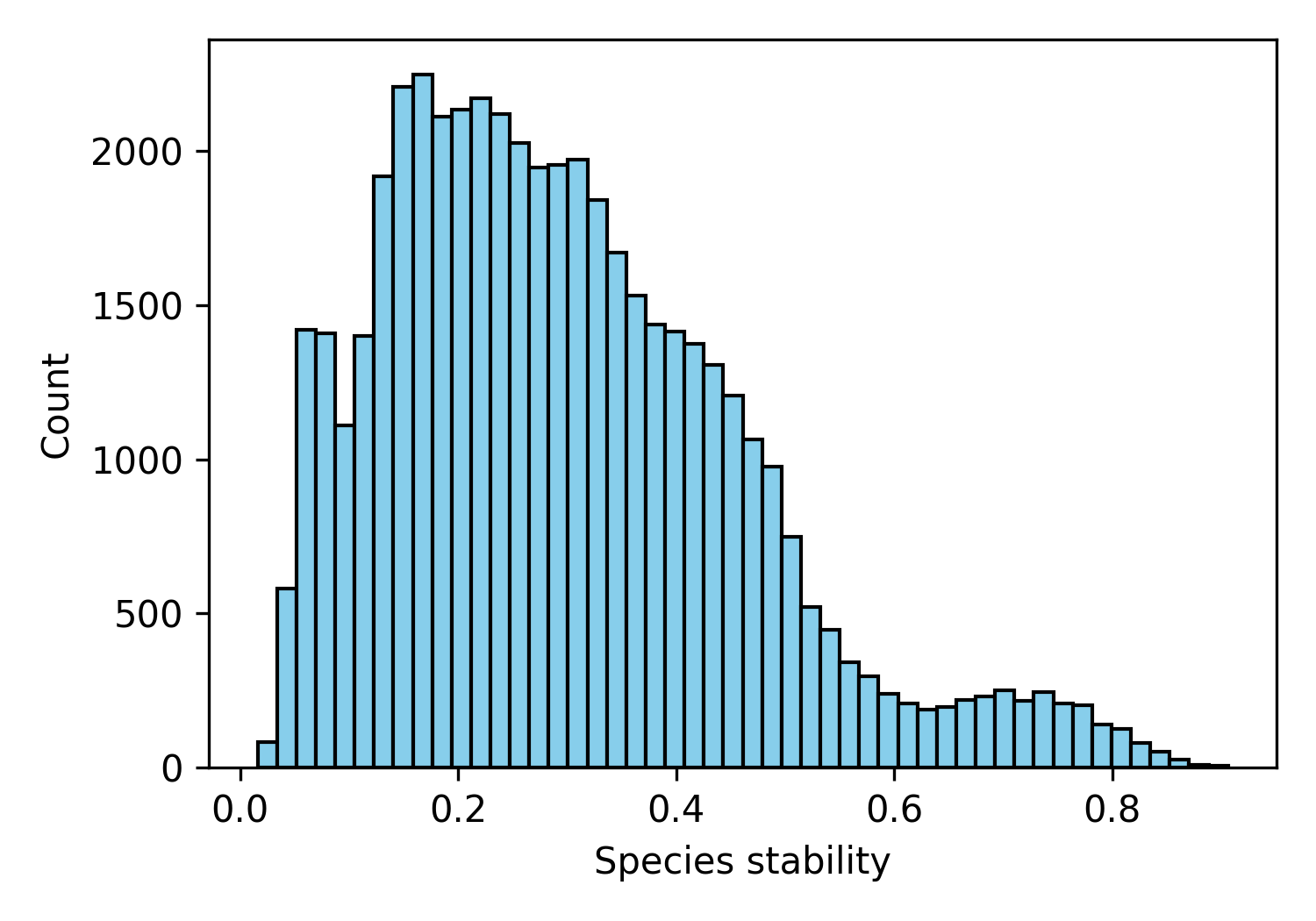

Supplement: S14 Fig — Histogram of species stability values computed from the consensus matrix, where stability is defined as the ratio between the average similarity of a species to members of its assigned cluster and its average similarity to all species. Higher values indicate more consistent co-clustering across resamples. (PNG) [file pcbi.1014278.s017.png]

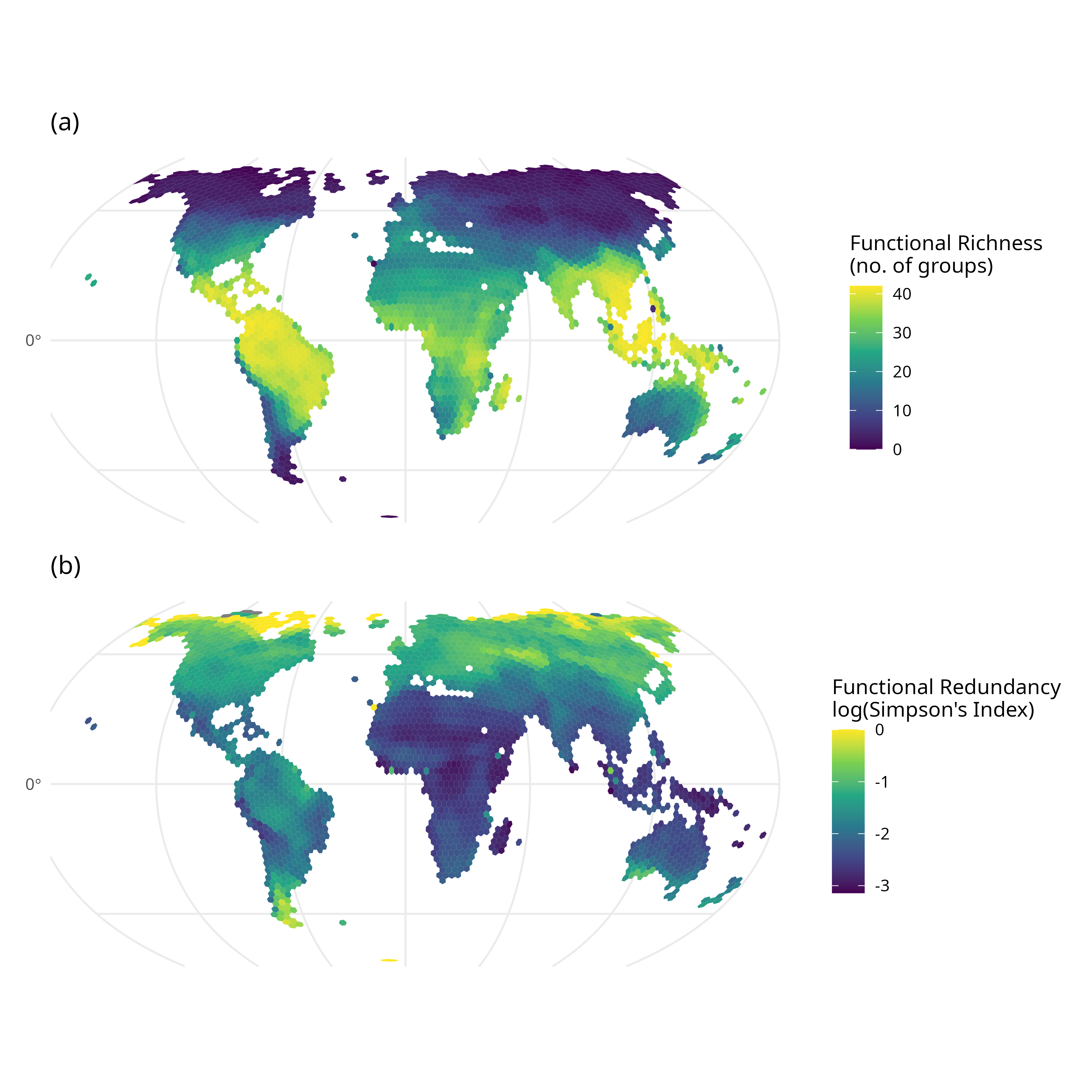

Supplement: S15 Fig — (a) Functional Richness is the number of functional groups per pixel (200 km nominal diameter hexagonal bins), and (b) Functional Redundancy is Simpson’s Index applied to the functional groups, here shown on the log scale for better spatial resolution. Compare to the results of Paz et al. [12]. (PNG) [file pcbi.1014278.s018.png]
