# Supplementary material for: Functional group classification using consensus clustering
Source: PLoS Comput Biol. 2026 May 13;22(5):e1014278. doi: 10.1371/journal.pcbi.1014278 (PMC13197079; doi:10.1371/journal.pcbi.1014278)
Supplement: S1 Fig — Correlation matrix for all 18 traits (after natural log), with a color scale where blue denotes positive correlations and red signifies negative correlations. Most traits exhibit significant correlations, highlighting the necessity of accounting for these relationships in clustering and distance analyses. (PDF) [file pcbi.1014278.s004.pdf]

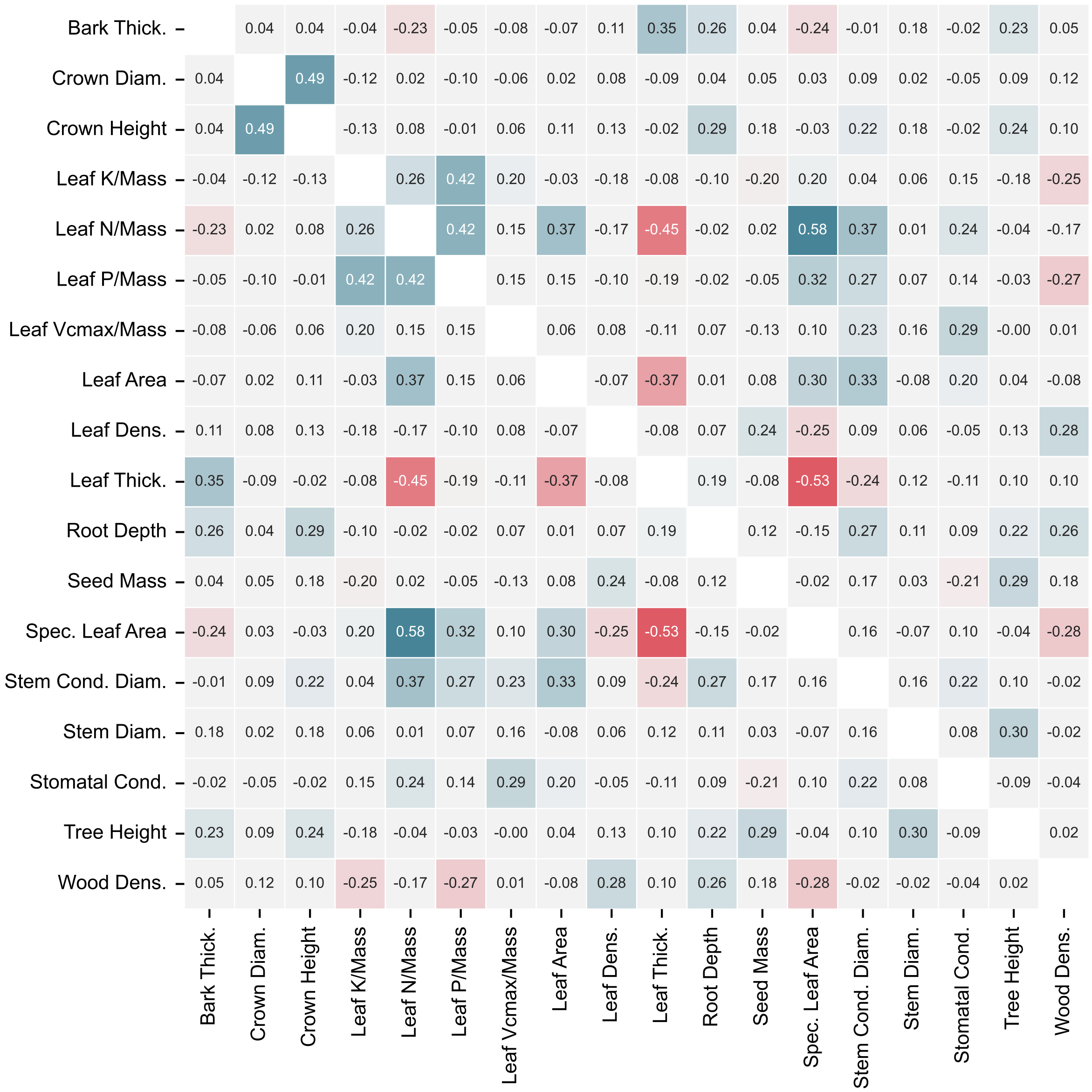

Bark Thick. 0.04 0.04 -0.04 -0.23 -0.05 -0.08 -0.07 0.11 0.35 0.26 0.04 -0.24 -0.01 0.18 -0.02 0.23 0.05
Crown Diam. 0.04 0.49 -0.12 0.02 -0.10 -0.06 0.02 0.08 -0.09 0.04 0.05 0.03 0.09 0.02 -0.05 0.09 0.12
Crown Height 0.04 0.49 -0.13 0.08 -0.01 0.06 0.11 0.13 -0.02 0.29 0.18 -0.03 0.22 0.18 -0.02 0.24 0.10
Leaf K/Mass -0.04 -0.12 -0.13 0.26 0.42 0.20 -0.03 -0.18 -0.08 -0.10 -0.20 0.20 0.04 0.06 0.15 -0.18 -0.25
Leaf N/Mass -0.23 0.02 0.08 0.26 0.42 0.15 0.37 -0.17 -0.45 -0.02 0.02 0.58 0.37 0.01 0.24 -0.04 -0.17
Leaf P/Mass -0.05 -0.10 -0.01 0.42 0.42 0.15 0.15 -0.10 -0.19 -0.02 -0.05 0.32 0.27 0.07 0.14 -0.03 -0.27
Leaf Vcmax/Mass -0.08 -0.06 0.06 0.20 0.15 0.15 0.06 0.08 -0.11 0.07 -0.13 0.10 0.23 0.16 0.29 -0.00 0.01
Leaf Area -0.07 0.02 0.11 -0.03 0.37 0.15 0.06 -0.07 -0.37 0.01 0.08 0.30 0.33 -0.08 0.20 0.04 -0.08
Leaf Dens. 0.11 0.08 0.13 -0.18 -0.17 -0.10 0.08 -0.07 -0.08 0.07 0.24 -0.25 0.09 0.06 -0.05 0.13 0.28
Leaf Thick. 0.35 -0.09 -0.02 -0.08 -0.45 -0.19 -0.11 -0.37 -0.08 0.19 -0.08 -0.53 -0.24 0.12 -0.11 0.10 0.10
Root Depth 0.26 0.04 0.29 -0.10 -0.02 -0.02 0.07 0.01 0.07 0.19 0.12 -0.15 0.27 0.11 0.09 0.22 0.26
Seed Mass 0.04 0.05 0.18 -0.20 0.02 -0.05 -0.13 0.08 0.24 -0.08 0.12 -0.02 0.17 0.03 -0.21 0.29 0.18
Spec. Leaf Area -0.24 0.03 -0.03 0.20 0.58 0.32 0.10 0.30 -0.25 -0.53 -0.15 -0.02 0.16 -0.07 0.10 -0.04 -0.28
Stem Cond. Diam. -0.01 0.09 0.22 0.04 0.37 0.27 0.23 0.33 0.09 -0.24 0.27 0.17 0.16 0.16 0.22 0.10 -0.02
Stem Diam. 0.18 0.02 0.18 0.06 0.01 0.07 0.16 -0.08 0.06 0.12 0.11 0.03 -0.07 0.16 0.08 0.30 -0.02
Stomatal Cond. -0.02 -0.05 -0.02 0.15 0.24 0.14 0.29 0.20 -0.05 -0.11 0.09 -0.21 0.10 0.22 0.08 -0.09 -0.04
Tree Height 0.23 0.09 0.24 -0.18 -0.04 -0.03 -0.00 0.04 0.13 0.10 0.22 0.29 -0.04 0.10 0.30 -0.09 0.02
Wood Dens. 0.05 0.12 0.10 -0.25 -0.17 -0.27 0.01 -0.08 0.28 0.10 0.26 0.18 -0.28 -0.02 -0.02 -0.04 0.02
Bark Thick.
Crown Diam.
Crown Height
Leaf K/Mass
Leaf N/Mass
Leaf P/Mass
Leaf Vcmax/Mass
Leaf Area
Leaf Dens.
Leaf Thick.
Root Depth
Seed Mass
Spec. Leaf Area
Stem Cond. Diam.
Stem Diam.
Stomatal Cond.
Tree Height
Wood Dens.
